# Factors influencing workplace violence among Chinese nurses: a multicenter cross-sectional study

**DOI:** 10.3389/fpubh.2025.1697768

**Published:** 2025-12-15

**Authors:** Xuehui Liu, Wei Zheng, Xinru Liu

**Affiliations:** 1 Department of Cardiology, West China Hospital, Sichuan University/West China School of Nursing, Sichuan University, Chengdu, Sichuan, China; 2 Department of Obstetrics and Gynaecology Clinic Nursing, West China Second University Hospital, Sichuan University/West China School of Nursing, Sichuan University, Chengdu, Sichuan, China; 3 Key Laboratory of Birth Defects and Related Diseases of Women and Children, Ministry of Education, Sichuan University, Chengdu, Sichuan, China

**Keywords:** mental health, WPV, prevalence, risk factor, protective factor, nurses, China

## Abstract

**Background:**

Workplace violence against nurses and nursing students is a pervasive global issue. A comprehensive understanding of its influencing factors is imperative for developing effective prevention strategies. This study aimed to conduct a thorough examination of the incidence and contributing factors of WPV and its subtypes among nurses undergoing standardized training in Sichuan Province, China.

**Methods:**

A large-scale, multicenter cross-sectional study was conducted using an anonymous online questionnaire. A cohort of nurses in standardized training from 90 hospitals across Sichuan Province was invited to participate. Data were analyzed using descriptive statistics and binary logistic regression models.

**Results:**

The study included 7,231 nurses in standardized training, of whom 1,328 (18.37%) reported experiencing WPV. The prevalence of WPV subtypes was as follows: emotional abuse (15.72%), threats (7.01%), verbal assault (4.98%), physical assault (3.84%), and sexual assault (2.50%). Binary logistic regression analysis identified several protective factors against personally experienced WPV, including being female (OR = 0.690), living with parents (OR = 0.825), perceiving career prospects as better than the current level (OR = 0.714), higher job-related social support (OR = 0.846), greater psychological resilience (OR = 0.837), stronger professional identity (OR = 0.845), higher bullying scores (OR = 0.865), and witnessing verbal assault against colleagues (OR = 0.582). Conversely, risk factors for WPV included living in an urban area (OR = 1.185), holding religious beliefs (OR = 1.562), having experienced occupational exposure (OR = 1.386), caring for more than 12 patients during a day shift (OR = 1.359), personal experience with medical complaints (OR = 2.098), higher adaptive performance scores (OR = 1.141), and witnessing WPV against colleagues (OR = 38.490).

**Conclusion:**

The prevalence of WPV against nurses in standardized training in China was lower than rates reported in other studies, yet the issue remains significant. Comprehensive strategies are needed to improve work environments and support the mental health of nurses, which may effectively reduce the incidence of WPV.

## Background

Workplace violence (WPV) is defined as “incidents where staff are abused, threatened or assaulted in circumstances related to their work, including commuting to and from work, involving an explicit or implicit challenge to their safety, wellbeing or health.” It is a pervasive issue within healthcare systems globally (1). A systematic review revealed that 61.9% of healthcare workers worldwide have encountered WPV at some point in their careers (2), with nurses being particularly vulnerable due to their frontline roles (3, 4). Furthermore, nursing students are also highly susceptible to WPV, primarily due to their limited clinical experience, frequent clinical rotations, and the challenge of establishing rapport with patients and multidisciplinary teams within a short timeframe (5). A multicenter cross-sectional study in China reported that 42.98% of nursing interns experienced at least one violent event in the past year, including verbal abuse (38.47%) (6). These findings are consistent with other research. For instance, one survey revealed that in medical settings, threats, physical attacks, sexual harassment, and disruptive medical disputes accounted for 14.78, 2.73, 1.99, and 1.78% of WPV incidents, respectively. Recent studies have also found that 27.23% of newly graduated nurses experienced violence from colleagues, and 56.17% witnessed it (7). To facilitate the professional integration of new nursing graduates, the Chinese government introduced the “Training Outline for Newly Recruited Nurses (Trial Implementation)” in 2016, which recommends a 24-month standardized training program in tertiary hospitals. However, the prevalence of WPV within this specific cohort has not been fully explored.

The consequences of WPV are severe and multifaceted, affecting staff wellbeing, job efficacy, and patient care quality. Exposure to WPV is associated with a heightened risk of depression, anxiety, post-traumatic stress disorder (PTSD), and suicidal ideation among healthcare professionals (8, 9). It has also been linked to burnout symptoms among physicians and nurses (10). The emotional distress following WPV incidents can lead to an increased risk of medical errors and a decline in the quality of care (11). Studies have shown that psychiatric nurses who experience WPV often report a significant decrease in their quality of life, health satisfaction, and career satisfaction, alongside a greater intention to leave their jobs (12). Therefore, addressing WPV is critical not only for healthcare workers but also for maintaining a safe and effective healthcare system.

Existing evidence highlights multiple individual and organizational factors associated with WPV among healthcare workers. A systematic review of 139,533 healthcare workers in intensive care units across 32 countries found that younger age and less work experience were risk factors for WPV (13). Similar patterns were observed among psychiatric nurses, were male gender and younger age increased vulnerability (12). At the institutional level, a study in primary hospitals identified demographic characteristics (gender, age, marital status, education) and hospital size (number of beds) as independent predictors of WPV risk (14). Another study involving 2,769 Chinese nurses further revealed that occupational stressors (night shifts, adverse care events), health status (chronic diseases, sleep disorders), and coping styles (passive coping) were risk factors for WPV, while better working conditions and active coping served as protective factors (15). Notably, a study of 1,015 general practitioners demonstrated distinct risk patterns by violence type, with physical violence linked to male gender and non-physical violence correlated with administrative roles (16). Despite these findings, significant gaps remain in understanding the psychological determinants of WPV, such as psychological resilience, adaptability, and professional identity.

Therefore, this multicenter study aimed to comprehensively investigate the multifactorial determinants of WPV among nurses in standardized training in China. We integrated four critical dimensions into our analysis: (i) demographic characteristics, (ii) working conditions, (iii) lifestyle factors, and (iv) mental and physical health. Our study seeks to provide robust evidence to inform the development of multidimensional strategies tailored to prevent WPV for this important group of early-career nurses.

Based on the existing literature gaps, this study aimed to address the following research questions:

1. What is the prevalence of WPV and its subtypes among nurses in standardized training in China?
2. What factors are associated with WPV exposure in this population?
3. Do protective and risk factors differ across WPV subtypes (physical assault, emotional abuse, threats, verbal assault, and sexual assault)?

## Methods

### Study design

This large-scale, multicenter cross-sectional study recruited volunteer nurses from 90 hospitals in Sichuan Province, China, between March 2023 and March 2024.

### Search methods

The questionnaire was distributed to study participants through multiple channels, including WeChat groups, direct messaging, QR code scanning at onsite locations, and distribution at hospitals. The detailed questionnaire is provided in Supplementary material 1.

Inclusion criteria were: (i) being enrolled in a Standardized Residency Training Program for Nurses in Sichuan Province; (ii) having a primary training placement within Sichuan Province; (iii) understanding the study’s purpose and voluntarily consenting to participate; (iv) possessing sufficient literacy to complete the questionnaire independently; and (v) being actively enrolled in training during the data collection period.

Exclusion criteria were: (i) not being enrolled in a standardized training program (e.g., visiting nurses); (ii) being absent from training for over 1 month during the study period; (iii) refusing to provide informed consent; (iv) having a missing data rate exceeding 10% for key variables; (v) having a known mental or cognitive impairment affecting response accuracy; and (vi) having previously participated in a pre-survey for this study.

### Ethical approval and consent inform

Ethical approval was obtained from the Ethics Committee of West China Hospital (Approval No. 2023822). As the survey was conducted online, participants provided digital informed consent by clicking a confirmation box on the first page of the questionnaire. They were informed that participation was voluntary, confidential, and that they could withdraw at any time.

### Dependent outcome variables and instruments

The primary dependent outcome was the experience of WPV. Participants were asked if they had personally experienced or witnessed a colleague experience WPV in the past year. Affirmative responses prompted further questions about specific types of violence: physical assault, emotional abuse, threats, verbal assault, and sexual assault. For physical health, participants reported any physician-diagnosed chronic diseases.

### Independent outcome variables and instruments

The questionnaire collected data across four domains:

- *Demographic information*: Gender, age, region, education level, monthly family and personal income, marital status, religious beliefs, household size, and childhood “left behind” experience.
- *Working conditions*: Hospital level, history of occupational exposure, weekly work hours, shift frequency and duration, number of patients cared for per day shift, patient deaths in the last 6 months, and daily commute time. Nurse–patient relationship satisfaction was assessed by personal or observed experience with medical complaints. Job satisfaction was measured on a 5-point Likert scale (very unsatisfied to very satisfied). Perceived career prospects were categorized as worse, unchanged, or better than the current level. The Job Content Questionnaire (JCQ) was used to assess job demands (five items), job control (nine items), and job social support (eight items) (17). Each item was rated on a 5-point Likert scale, with higher scores indicating greater job demands, higher job control, and stronger social support. Job Content Questionnaire (JCQ): Job demands: “My job requires working very fast”; Job control: “My job allows me to make a lot of decisions on my own”; Social support: “My supervisor is helpful in getting the job done.”
- *Lifestyles factors*: Sleep was assessed by duration, quality, and napping habits. Alcohol consumption was defined as drinking at least twice a week for over 6 months, and smoking was defined as smoking at least one cigarette daily for over 6 months. Exercise was evaluated by habit, frequency, and duration.
- *Mental and physical health metrics*: Multiple questionnaires were used to collect a comprehensive situation of participants in the Mental and Physical Health Metrics section of the questionnaire:

*Psychological resilience* was assessed using the *Chinese version of the Resilience Scale-14*, which has demonstrated high reliability among Chinese junior nurses (18). The scale consists of 14 items, divided into two factors: Factor 1 reflects personal competence, while Factor 2 reflects acceptance of oneself and life (19). This scale utilizes a 5-point scoring method, ranging from 1 (strongly disagree) to 5 (strongly agree). The total score ranges from 14 to 70 points, with higher scores indicating better psychological resilience (19). Resilience Scale-14 (RS-14): Personal competence: “I usually manage one way or another”; for acceptance of self and life: “I can get through difficult times because I’ve experienced difficulty before.”

*Adaptive performance* was measured using the *Adaptive Performance Scale (APS)* developed by Tao et al. (20). The scale consists of 15 items, and 13 items focused exclusively on two dimensions closely related to the adaptive behaviors of nurses were selected to be analyzed in our study: (i) stress and emergency handling (factor 1), and (ii) interpersonal and cultural adaptation (factor 2). A 5-point Likert scale was used, with total scores ranging from 13 to 65, with higher scores indicating better adaptability performance. Adaptive Performance Scale (APS): Stress/emergency handling: “I can quickly adjust my work methods when facing sudden work pressure”; Interpersonal/cultural adaptation: “I can effectively cooperate with colleagues from different backgrounds.”

*Social support* was measured using the *Social Support Rating Scale (SSRS)* designed by Xiao. The scale comprises three dimensions: objective support (three items, sum of items 2, 6, and 7), subjective support (four items, sum of items 1, 3, 4, and 5), and utilization of social support (three items, sum of items 8, 9, and 10). The scoring method for each item varies, and a higher total score indicates a greater level of perceived social support (<20 indicates low social support, 20–30 indicates moderate social support, and>30 indicates high social support) (21). Social Support Rating Scale (SSRS): Objective support: “How many close friends do you have who can provide help and support?”; Subjective support: “How much concern and care do you receive from your family?”

*Professional identity* was evaluated using the *Professional Identity Scale for Nurses (PISN)* developed by Liu et al. (22) specifically for nurses. The Professional Identity Scale comprises nine items, each rated on a 5-point Likert scale, with a higher score reflecting a stronger sense of professional identity. Professional Identity Scale for Nurses (PISN): “I am proud to be a nurse,” “I believe nursing is a valuable profession for society.”

For the measurement of *Bullying*, we employed the *Negative Acts Questionnaire (NAQ)* translated by Peng (23). To reduce respondent burden within a comprehensive questionnaire, we selected six items from the original 22-item NAQ that were most relevant to workplace dynamics in a clinical nursing context, scored on a scale from 1 (very rarely) to 5 (very frequently) (23), with higher total scores indicating a greater frequency of exposure to this type of bullying behaviors. The abbreviated 6-item NAQ scale demonstrated good internal consistency in our sample, with a Cronbach’s alpha of 0.86. Negative Acts Questionnaire (NAQ): “Being humiliated or ridiculed in connection with your work” and “Being exposed to an unmanageable workload.”

### Statistical analysis

The statistical analysis was performed using STATA 23.0. The continuous variables were tested for normal distribution using the Kolmogorov–Smirnov test. Continuous data with a normal distribution were described as mean ± standard deviation (SD) and analyzed using a *t*-test. Categorical data were described as *n* (%) and analyzed using the chi-square test. Two-sided *p*-values < 0.05 were considered statistically significant. To identify factors associated with self-involving WPV (yes = 1, no = 0), a binary logistic regression model was constructed with self-involving WPV as the dependent variable and candidate predictors as independent variables. To further investigate the determinants of each WPV subtype (yes = 1, no = 0), multivariable ordered logistic regression models were fitted, with the frequency of exposure to each subtype as the dependent variable. Post-estimation diagnostics were performed to ensure model validity. Multicollinearity was assessed using the Variance Inflation Factor (VIF), and all VIF values were found to be below 5, indicating that multicollinearity was not a significant concern in our model.

## Results

### Participant recruitment

**Figure 1:**
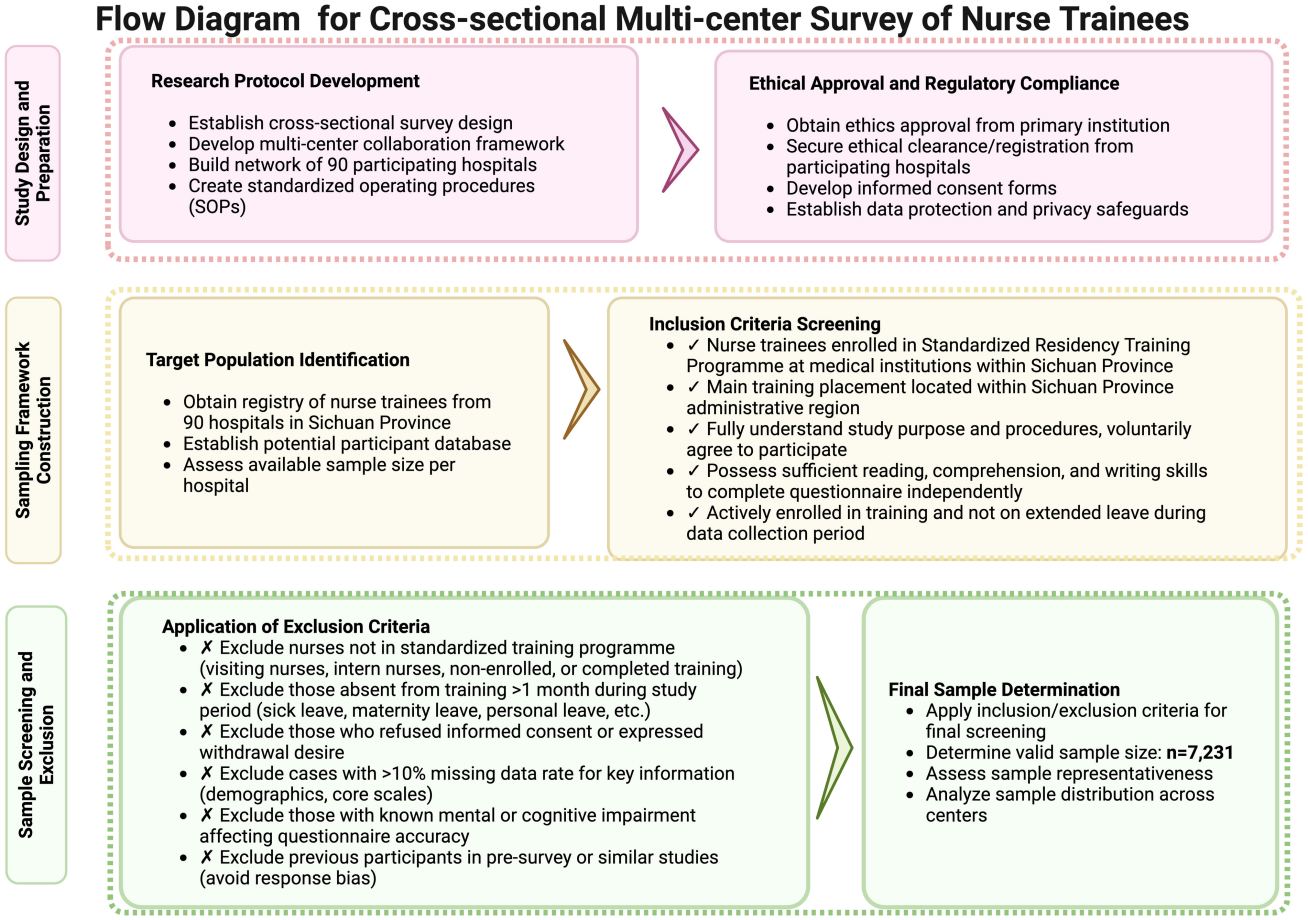
The flow diagram for our study.

A total of 7,231 participants were included in the final analysis. Figure 1 presents the flow chart detailing participant recruitment and inclusion.

### Demographic characteristics of participants

Table 1 displays the demographic characteristics of the participants. The majority were female (92.86%, *n* = 6,715), with a mean age of 23.02 years (SD = 1.62). Nearly a quarter (24.81%) held a bachelor’s degree or higher. The mean monthly personal income was 2,614.63 CNY (SD = 1142.76). Most participants were single (91.56%). A small percentage held religious beliefs (5.68%), while approximately half lived with their parents (51.17%) and had a childhood “left behind” experience (50.21%).

**Table 1:** Characteristics of participants in this study (*N* = 7,231).

Variables	*n* (%)
Demographic characteristics	
Gender	
Male	516 (7.14)
Female	6,715 (92.86)
Age^a^	23.023 (1.62)
Region	
Urban	2,017 (27.89)
Rural	5,214 (72.11)
Education level	
Vocational high school	179 (2.48)
Two-year college	5,258 (72.71)
Bachelor’s degree or above	1,794 (24.81)
Family income per month (CNY)^a^	3638.75 (3799.41)
Own income per month (CNY)^a^	2614.63 (1142.76)
Marital status	
Single	6,621 (91.56)
Married	579 (8.01)
Others	31 (0.43)
Religious beliefs	
Yes	411 (5.68)
No	6,820 (94.32)
Number of household members^a^	3.86 (1.26)
Living with parents	
Yes	3,700 (51.17)
No	3,531 (48.83)
Experience of being left behind	
Yes	3,631 (50.21)
No	3,600 (49.79)
Working conditions	
Hospital level	
Tertiary III	6,213 (85.92)
Non-tertiary III	1,018 (14.08)
Occupational exposure	
Yes	3,515 (48.61)
No	3,716 (51.39)
Working length (hours/week)	
Within 40 h	2,085 (28.83)
41–55 h	4,195 (58.01)
56–70 h	754 (10.43)
More than 70 h	197 (2.72)
Day shift frequency per week^a^	3.48 (1.15)
Day shift length (hours)^a^	8.20 (0.75)
Night shift frequency per week^a^	1.86 (0.80)
Night shift length (hours)^a^	8.36 (1.28)
Number of patients cared for during a day shift^a^	9.37 (6.00)
Number of deaths after cared in the last 6 months^a^	1.15 (2.72)
Daily commuting time to work (hours)^a^	0.92 (0.56)
Own medical complaints	
Yes	214 (2.96)
No	7,017 (97.04)
Medical complaints of colleagues	
Yes	3,233 (44.71)
No	3,998 (55.29)
Feel a balance between effort and return	
Very unsatisfied	428 (5.92)
Unsatisfied	1,183 (16.36)
Neutral	3,205 (44.32)
Satisfied	2,115 (29.25)
Very satisfied	300 (4.15)
Career prospects	
Below current level	492 (6.80)
Unchanged	2,009 (27.78)
Higher than current level	4,730 (65.41)
Job demands^a^	18.05 (2.83)
Job control^a^	32.15 (4.14)
Job social support^a^	29.62 (5.13)
Degree of job stress^a^	1.01 (0.12)
Lifestyles	
Sleep duration (hours per day)^a^	6.92 (0.86)
Sleep quality	
Good	1,598 (22.10)
Normal	4,463 (61.72)
Bad	1,170 (16.18)
Nap at lunchtime	
Yes	4,992 (69.04)
No	2,239 (30.96)
Alcohol consumption	
Yes	154 (2.13)
No	7,077 (97.87)
Smoking	
Yes	122 (1.69)
No	7,109 (98.31)
Exercise habits	
Yes	2,519 (34.84)
No	4,712 (65.16)
Exercise frequency (per week)	
1–2 times	6,649 (91.95)
3–4 times	470 (6.50)
>5 times	112 (1.55)
Exercise duration (min per time)^a^	14.49 (27.88)
Mental and physical health	
Resilience (total score)^a^	49.49 (8.32)
Resilience (factor 1)^a^	34.84 (6.09)
Resilience (factor 2)^a^	14.65 (2.49)
Adaptive performance^a^	226.84 (26.14)
Social support^a^	8.13 (1.86)
Professional identity^a^	32.93 (5.71)
Bullying^a^	12.93 (4.76)
Chronic disease	
Yes	6,999 (96.79)
No	232 (3.21)
Hypertension	
Yes	10 (0.14)
No	7,221 (99.86)
Asthma	
Yes	11 (0.15)
No	7,220 (99.85)
Diabetes	
Yes	11 (0.15)
No	7,220 (99.85)
Others	
Yes	213 (2.95)
No	7,018 (97.05)

a Present as means and standard deviations.

### Prevalence of workplace violence

Among the 7,231 participants, 18.37% (*n* = 1,328) had experienced WPV in the past year. The most common form was emotional abuse (15.72%), followed by threats (7.01%), verbal assault (4.98%), physical assault (3.84%), and sexual assault (2.50%). Additionally, 34.01% (*n* = 2,459) had witnessed colleagues experiencing WPV, with emotional abuse being the most frequently observed type (31.09%). The frequency of exposure to each type of WPV is detailed in Table 2.

**Table 2:** The prevalence of self-involving WPV and witness of colleagues’ WPV.

Variables	*n*	%
Self-involving WPV		
Experience WPV^a^		
Yes	1,328	18.37
No	5,903	81.63
Physical assault		
Never	6,953	96.16
Once	197	2.72
Sometimes	67	0.93
Often	14	0.19
Emotional abuse		
Never	6,094	84.28
Once	415	5.74
Sometimes	471	6.51
Often	251	3.47
Threatening		
Never	6,724	92.99
Once	327	4.52
Sometimes	133	1.84
Often	47	0.65
Verbal assault		
Never	6,871	95.02
Once	244	3.37
Sometimes	90	1.24
Often	26	0.36
Sexual assault		
Never	7,050	97.50
Once	128	1.77
Sometimes	45	0.62
Often	8	0.11
Witness of Colleagues’ WPV		
Experience WPV^a^		
Yes	2,459	34.01
No	4,772	65.99
Physical assault		
Never	5,932	82.04
Once	856	11.84
Sometimes	354	4.90
Often	89	1.23
Emotional abuse		
Never	4,983	68.91
Once	700	9.68
Sometimes	890	12.31
Often	658	9.10
Threatening		
Never	5,905	81.66
Once	719	9.94
Sometimes	423	5.85
Often	184	2.54
Verbal assault		
Never	6,437	89.02
Once	453	6.26
Sometimes	240	3.32
Often	101	1.40
Sexual assault		
Never	6,704	92.71
Once	348	4.81
Sometimes	134	1.85
Often	45	0.62

a Experience WPV indicates that experience any types of WPV, including physical assault, emotional abuse, threatening, verbal assault, and sexual assault.

WPV, workplace violence.

### Comparison between groups with and without workplace violence

A comparison of demographic characteristics, working conditions, lifestyle factors, and health metrics between standard training nurses who had and had not experienced WPV is provided in Supplementary Table 1.

### Factors associated with personally experienced workplace violence

A multivariable logistic regression analysis was conducted to identify factors associated with WPV. The variable assignments are shown in Supplementary Table 2.

As shown in Table 3, the analysis identified several protective factors: being female (OR = 0.690), living with parents (OR = 0.825, *p* = 0.028), perceiving career prospects as better than the current level (OR = 0.714, *p* = 0.029), higher job-related social support (OR = 0.846, *p* < 0.001), greater psychological resilience (OR = 0.837, *p* < 0.001), stronger professional identity (OR = 0.845, *p* < 0.001), higher bullying scores (OR = 0.865, *p* < 0.001), and witnessing verbal assault against colleagues (OR = 0.582, *p* < 0.001).

**Table 3:** Predicting protective and risk factors of WPV by Logistic model.

Variables	*β*	SE	OR	95% CI of OR		*p-*value
				lower	upper	
Demographic characteristics						
Gender						
Female	0.109	−2.360	0.690*	0.507	0.940	**0.019**
Age	0.029	−1.210	0.964	0.909	1.023	0.228
Region of hospital						
Urban	0.115	1.740	1.185	0.979	1.434	0.081
Education level						
Two-year college	0.238	−0.210	0.948	0.579	1.551	0.831
Postgraduate	0.251	−0.220	0.943	0.560	1.589	0.827
Marital status						
Married	0.313	−1.150	0.439	0.109	1.778	0.249
Others	0.347	−1.000	0.497	0.127	1.955	0.317
Religious belief						
Yes	0.258	2.690	1.562**	1.129	2.160	**0.007**
Number of household members						
One person	0.294	0.360	1.100	0.651	1.859	0.720
Two persons	0.225	−0.340	0.921	0.571	1.488	0.737
>2 persons	0.206	−0.590	0.869	0.545	1.384	0.553
Living with parents						
Yes	0.072	−2.200	0.825*	0.695	0.979	**0.028**
Experience of being left behind						
Yes	0.100	1.450	1.136	0.957	1.350	0.146
Working conditions						
Hospital level						
Tertiary III	0.120	−0.430	0.947	0.739	1.215	0.671
Occupational exposure						
Yes	0.120	3.780	1.386**	1.170	1.642	**0.000**
Working length (hours/week)						
41–55 h	0.107	0.460	1.048	0.858	1.280	0.649
56–70 h	0.179	0.720	1.122	0.821	1.533	0.470
More than 70 h	0.241	−0.210	0.947	0.576	1.558	0.831
Own income per month						
Below China’s national average disposable income	0.175	−1.600	0.651	0.384	1.102	0.110
Above China’s national average disposable income	0.208	−0.930	0.780	0.462	1.315	0.350
Day shift frequency per week						
15–22 days	0.130	0.800	1.100	0.872	1.387	0.421
>22 days	0.149	0.370	1.054	0.799	1.391	0.710
Day shift length (hours)	0.058	−0.620	0.963	0.855	1.084	0.533
Night shift frequency per month						
2–6 times	0.103	0.260	1.027	0.843	1.250	0.795
>6 times	0.164	0.930	1.142	0.863	1.512	0.353
Night shift length	0.037	1.100	1.040	0.970	1.114	0.271
Caring patients (no.)						
5–8	0.158	1.180	1.173	0.901	1.528	0.236
9–12	0.149	1.310	1.180	0.922	1.510	0.189
>12	0.168	2.480	1.359*	1.067	1.732	**0.013**
Patients’ death (no.)						
1	0.140	0.550	1.075	0.833	1.386	0.580
>1	0.100	0.330	1.032	0.853	1.248	0.745
Daily commuting time to work (hours)	0.077	1.110	1.082	0.941	1.244	0.267
Own medical complaints						
Yes	0.420	3.700	2.098**	1.417	3.106	**0.000**
Medical complaints of colleagues						
Yes	0.079	−1.690	0.857	0.716	1.026	0.092
Feel a balance between effort and return						
Unsatisfied	0.748	0.130	1.094	0.286	4.176	0.896
Neutral	0.438	−0.450	0.775	0.256	2.344	0.652
Satisfied	0.356	−0.820	0.632	0.210	1.904	0.414
Very satisfied	0.243	−1.500	0.414	0.131	1.311	0.134
Career prospects						
Unchanged	0.143	−0.670	0.899	0.657	1.229	0.504
Higher than current level	0.110	−2.190	0.714*	0.528	0.966	**0.029**
Job demands	0.102	−1.610	0.818	0.641	1.045	0.108
Job control	0.063	−1.800	0.879	0.764	1.011	0.071
Job social support	0.014	−10.140	0.846**	0.819	0.874	**0.000**
Degree of job stress	3.724	0.280	1.781	0.030	107.337	0.783
Lifestyles						
Nap at lunchtime						
Yes	0.104	1.180	1.117	0.930	1.341	0.238
Alcohol consumption						
Yes	0.227	−0.520	0.874	0.525	1.456	0.605
Smoking						
Yes	0.302	0.070	1.020	0.572	1.821	0.946
Exercise habits						
Yes	0.187	−0.690	0.860	0.561	1.318	0.490
Exercise frequency (per week)						
3–4 times	0.162	−0.470	0.920	0.651	1.300	0.637
Exercise duration (min/time)	0.003	0.840	1.002	0.997	1.007	0.399
Mental and physical health						
Resilience	0.012	−12.260	0.837**	0.814	0.862	**0.000**
Adaptive performance	0.011	14.200	1.141**	1.121	1.162	**0.000**
Social support	0.026	1.460	1.037	0.987	1.090	0.145
Professional identity	0.013	−11.040	0.845**	0.820	0.871	**0.000**
Bullying	0.012	−10.350	0.865**	0.841	0.889	**0.000**
Workplace violence exposure						
Witness of colleagues’ WPV						
Yes	5.818	24.150	38.490**	28.621	51.763	0.000
Witness of colleagues’ physical assault						
Yes	0.064	0.500	1.031	0.913	1.165	0.619
Witness of colleagues’ emotional abuse						
Yes	0.050	−1.440	0.925	0.832	1.028	0.149
Witness of colleagues’ threatening						
Yes	0.059	0.570	1.033	0.924	1.155	0.568
Witness of colleagues’ verbal assault						
Yes	0.043	−7.270	0.582**	0.503	0.674	0.000
Witness of colleagues’ sexual assault						
Yes	0.090	−0.450	0.959	0.798	1.152	0.655

Sensitivity 60.99%; specificity 93.43%; correctly classified 87.47%.

**p < 0.01, *p < 0.05.

China’s national average disposable income is 42,359 CNY per year, equals to 5,920 dollars.

Bold text denotes statistically significant results (*p* < 0.05).

Conversely, significant risk factors included: holding a religious belief (OR = 1.562, *p* = 0.007), having experienced occupational exposure (OR = 1.386, *p* < 0.001), caring for more than 12 patients during a day shift (OR = 1.359, *p* = 0.013), personal experience with medical complaints (OR = 2.098, *p* < 0.001), higher adaptive performance scores (OR = 1.141, *p* < 0.001), and witnessing WPV against colleagues (OR = 38.490, *p* < 0.001).

### Factors associated with subtypes of workplace violence

We further analyzed factors associated with the five subtypes of WPV. For physical assault, protective factors included being female (OR = 0.626, *p* = 0.043), living with parents (OR = 0.730, *p* = 0.038), higher psychological resilience (OR = 0.837, *p* < 0.001), stronger professional identity (OR = 0.852, *p* < 0.001), higher bullying scores (OR = 0.886, *p* < 0.001), and witnessing verbal or emotional abuse against colleagues. Risk factors included holding a religious belief (OR = 1.954, *p* = 0.005), working 2–6 night shifts per month (OR = 1.465, *p* = 0.040), caring for more than 12 patients per day shift (OR = 1.533, *p* = 0.044), higher adaptive performance (OR = 1.148, *p* < 0.001), and witnessing any WPV or physical assault against colleagues. Detailed results for all subtypes are available in Supplementary Tables 3–7.

## Discussion

### Prevalence of WPV among nurses in standardized training

Our study found that 18.37% of nurses in standardized training had experienced some form of WPV in the past year. This rate is lower than the prevalence reported in many domestic and international studies, which have shown rates ranging from 26.6 to 67.2% among nurses (15, 24–29). Dadfar and Lester (1) highlighted that WPV in healthcare systems remains a critical global concern requiring urgent attention and systematic intervention strategies. This lower prevalence may be attributable to several factors unique to standardized training programs. The clearly defined “learner” status of these nurses provides inherent protective mechanisms; their limited clinical responsibilities and close supervision by preceptors reduce direct exposure to high-risk conflict situations. Preceptors often act as a buffer between trainees and potential sources of violence.

However, this apparent protection may mask underlying issues of under-recognition and under-reporting. The most prevalent WPV subtypes in our sample—emotional abuse (15.72%), threats (7.01%), and verbal assault (4.98%)—are often subtle and ambiguous. The power dynamics inherent in training, where trainees depend on preceptors for evaluation, may lead to the normalization of non-physical violence or a reluctance to report incidents (30, 31). Therefore, the true prevalence of implicit violence may be underestimated, and sustained attention to protecting this cohort is essential.

### Demographic determinants

Our findings reveal significant gender-based differences, with males showing higher vulnerability to WPV overall (OR for being female = 0.690). This aligns with existing literature (32, 33) and may reflect complex sociocultural dynamics. Males entering a traditionally female-dominated profession might face challenges to their professional legitimacy, leading to confrontations. Societal stereotypes may also result in male nurses being assigned to manage aggressive patients more frequently, increasing their exposure.

Standardized training nurses with religious beliefs (OR = 1.562, *p* < 0.01) have higher risk of self-involved WPV, a finding consistent with Bagnasco et al. (34) and potentially attributable to value conflicts in clinical settings. This finding does not reflect religious belief itself, but rather potential value conflicts that can arise in diverse healthcare settings. Religious beliefs may create potential value conflicts or cultural misunderstandings in diverse healthcare environments, where nurses with strong religious convictions might face challenges when their personal beliefs conflict with certain medical procedures, patient care decisions, or institutional policies, potentially leading to tensions with patients, families, or colleagues who hold different worldviews.

Living with parents was a protective factor in our study. Parents, as stable confidants, a strong emotional support network, can help them alleviate stress in the face of conflict. Additionally, the presence of parental support creates a secure home environment that serves as a psychological refuge from workplace stressors, allowing them to decompress, process difficult experiences, and maintain better mental health, which in turn improves their ability to handle potentially volatile situations at work with greater composure and professional judgment.

### Organizational and workplace factors

Regarding organizational and workplace factors: occupational exposures, workloads (number of caring patients), personal experiences of medical complaints, perceived career prospects, job social support, and witnessing colleagues’ WPV were found to be significant factors for self-involving WPV event.

Occupational exposure experiences (OR = 1.386, *p* < 0.001) emerged as a risk factor in our study. Occupational exposure incidents such as needle stick injuries, radiation exposure, or chemical contamination can significantly increase standardized training nurses’ anxiety levels and create heightened stress responses that may impair their clinical judgment, communication skills, and ability to manage patient interactions effectively, potentially related to WPV. Additionally, standardized training nurses who have experienced occupational exposures may develop PTSD, hypervigilance, or fear-based behaviors that can affect their professional confidence and interpersonal relationships with patients, families, and colleagues, creating an atmosphere of tension that increases the likelihood of conflicts and aggressive encounters.

Consistent with established literature (14, 34), our study confirmed workload as a critical determinant of WPV exposure among standardized training nurses. Caring for more than 12 patients during day shifts was associated with significantly higher violence risk (OR = 1.359, *p* < 0.05), reflecting the relationship between excessive workload, reduced patient interaction time, and declining patient satisfaction scores.

Standardized training nurses having personal involvement in medical complaints (OR = 2.098, *p* < 0.001) are approximately twice as likely to encounter WPV compared to those without such experiences, reflects that cascading psychological and professional consequences of complaint involvement. Personal involvement in medical complaints can severely undermine their professional confidence, create persistent anxiety about clinical performance, and lead to defensive or overly cautious behaviors that may negatively affect patient communication and care delivery, potentially creating conditions that precipitate further conflicts with patients and families.

What is new to us is that the higher the participants perceived career prospects above current levels and the greater the social support at work (35), the less likely standardized training nurses were to experience WPV. Career advancement opportunities provide participants with a sense of purpose, foster professional growth, and offer future security, thereby reducing job-related stress and burnout that often contribute to vulnerability in high-stress clinical situations. Furthermore, robust social support systems create collaborative environments where standardized training nurses can seek assistance, share experiences, and receive guidance from colleagues and supervisors when facing difficult situations, thereby preventing conflict escalation and reducing individual exposure to violence. When they perceive strong institutional support for their professional development and career advancement, they are more likely to invest in developing effective communication skills, emotional regulation strategies, and conflict resolution techniques that serve as protective mechanisms against WPV.

Perhaps the most striking finding of our study is the exceptionally high odds ratio associated with witnessing colleagues experience WPV (OR = 38.49), identifying it as the most potent risk factor for personal WPV exposure. While such a high OR might initially suggest statistical artifacts like multicollinearity, our model diagnostics ruled out this concern, indicating a robust and meaningful association. We interpret this powerful finding as evidence of a significant “contagion effect” within the healthcare work environment. When acts of violence are witnessed and not met with swift, effective institutional responses, it can lead to a normalization of aggression in the workplace culture. This process fundamentally alters the psychological safety of all staff. This altered psychological state may change their communication patterns, potentially making them more defensive or less confident, which can inadvertently escalate tense situations. Therefore, witnessing WPV is not just a passive observation; it is a transformative experience that reshapes their interaction with environment, making standardized training nurses acutely sensitive and substantially more vulnerable to becoming a victim themselves.

### Mental and physical factors

In terms of mental and physical health, our study identified several critical psychological factors that is significantly correlated with WPV, which include psychological resilience, professional identity, adaptive performance, bullying.

Psychological resilience emerged as a robust protective factor (OR = 0.837, *p* < 0.001), consistent with theoretical frameworks suggesting that resilient individuals possess enhanced capacity for stress management, environmental adaptation, and supportive system utilization (36–38). This finding has important implications for recruitment, training, and ongoing professional development programs among standardized training nurses.

Professional identity demonstrated significant protective associations (OR = 0.845, *p* < 0.001) among standardized training nurses, reflecting the complex relationship between role clarity, professional confidence, and vulnerability to workplace aggression. Strong professional identity is associated with assertiveness, improved communication skills, foster respect from colleagues and patients, thereby reducing exposure to various forms of WPV. Our results are similar to those reported in published works by Olashore et al. (39) and Chang et al. (40). WPV disrupts the alignment between professional role expectations and obligations, thus are related to a lower level of professional identity of nurses (41).

Paradoxically, higher adaptive performance scores were associated with increased WPV (OR = 1.141, *p* < 0.001). Higher adaptive performance scores indicate that one can implement appropriate measures in emergency or crisis situations, managing work-related stress effectively, and integrating well within teams and the organization to form positive professional relationships (42). Possible explanations are that standardized training nurses with high adaptability may be assigned to manage more challenging and complex patients or higher-pressure clinical situations. Their supervisors may perceive them as more capable of handling these challenges, but this inadvertently increases their frequency and risk of exposure to conflict-prone environments.

Bullying exposure (OR = 0.865, *p* < 0.001) demonstrated significant protective associations. The counterintuitive finding can be explained by “psychological inoculation” or “threat recognition” hypothesis: bullying exposure acts as a protective factor may reflect a psychological inoculation effect, where prior exposure to lower-level workplace aggression enables standardized training nurses to develop enhanced coping strategies, threat recognition skills, and emotional regulation abilities that subsequently protect them from more severe forms of WPV. Additionally, standardized training nurses who have experienced bullying may be more likely to actively seek and establish stronger social support networks with colleagues and supervisors, creating protective buffers that reduce their vulnerability to future violent incidents through enhanced workplace relationships and collective support mechanisms. Furthermore, this finding may indicate that the bullying exposure scale in our study measured awareness and recognition of bullying behaviors rather than actual victimization experiences, suggesting that heightened sensitivity to workplace aggression patterns serves as an early warning system that enables proactive avoidance of potentially violent situations.

### Common protective factors across violence subtypes

Our analysis identified several protective factors that demonstrated remarkable consistency in reducing workplace violence risk across all subtypes, revealing important insights into the multifaceted nature of violence prevention in healthcare settings. (i) *Psychological resilience* emerged as the most potent and universal protective factor, consistently demonstrating odds ratios below 0.85 across all violence types (*p* < 0.001 for all). This robust finding highlights resilience as a cornerstone of violence prevention, suggesting that nurses with stronger psychological resources are better equipped to navigate challenging interpersonal situations, de-escalate conflicts, and maintain professional composure under stress. The consistency of this protective effect underscores the critical value of organizational investments in mental health resources and resilience-building programs as foundational elements of comprehensive violence prevention strategies. (ii) *Professional identity* exhibited similarly consistent protective benefits across all violence subtypes, with odds ratios ranging from 0.845 to 0.852 (all *p* < 0.001). This pattern suggests that nurses with stronger professional identity possess enhanced self-confidence, more effective communication skills, and superior conflict management capabilities that collectively reduce their vulnerability to violent encounters. The protective mechanism likely operates through improved patient interactions, clearer boundary-setting abilities, and greater assertiveness in challenging situations. (iii) *Family support systems*, as evidenced by living with parents, demonstrated notable protective effects across multiple violence types (odds ratios consistently below 0.80). This finding illuminates the crucial role that external social support networks play in violence prevention, suggesting that nurses with strong family connections benefit from enhanced emotional resources, stress management support, and psychological stability that translate into reduced workplace vulnerability. The protective effect underscores the importance of recognizing and supporting the broader social context within which nurses operate. (iv) *Gender differences* revealed a complex protective pattern, with female gender generally conferring protection across most violence subtypes, though notably not for verbal assault. This nuanced finding indicates the presence of gender-specific vulnerability patterns that reflect broader societal dynamics and workplace power structures, emphasizing the need for tailored intervention strategies that account for the differential ways in which violence manifests across gender lines in healthcare environments.

### Common risk factors across violence subtypes

Our analysis revealed several risk factors that demonstrated remarkable consistency across all WPV subtypes. Most notably, (i) *Witnessing colleagues’ WPV* emerged as the predominant risk factor, with odds ratios frequently exceeding 10.0 (*p* < 0.001 for all subtypes). This striking finding provides compelling evidence for contagion effects within healthcare environments, whereby exposure to colleagues’ victimization creates a cascading vulnerability that substantially elevates individual risk for subsequent violent encounters. (ii) *Religious beliefs* represented another universal risk factor, consistently elevating vulnerability across all violence subtypes (OR typically >1.5, *p* < 0.01). This pattern suggests the presence of underlying value conflicts or discriminatory attitudes within healthcare settings, underscoring the urgent need for comprehensive organizational interventions focused on cultural competency and inclusive workplace practices. (iii) *Occupational safety* incidents, including needle stick injuries and radiation exposures, demonstrated persistent associations with increased violence risk across all subtypes (OR typically >1.3, *p* < 0.001). This finding illuminates how workplace safety breaches create ripple effects that extend beyond immediate physical harm, potentially compromising nurses’ psychological wellbeing and interpersonal effectiveness in ways that heighten their susceptibility to violent encounters. (iv) *Workload-related factors*, particularly caring for more than 12 patients during day shifts, consistently elevated risk across violence subtypes (OR typically >1.3, *p* < 0.05). This relationship underscores the fundamental importance of adequate staffing ratios as a cornerstone of violence prevention strategies, highlighting how resource constraints create conditions conducive to patient dissatisfaction and aggressive behaviors. Perhaps most concerning, (v) *Personal involvement in medical complaint* processes demonstrated robust associations with violence risk across all subtypes (OR typically >2.0, *p* < 0.001). This finding suggests that complaint involvement creates a state of heightened vulnerability that persists beyond the immediate complaint resolution, necessitating targeted support interventions for affected nurses. Intriguingly, (vi) *Higher adaptive performance scores* paradoxically increased violence risk across all subtypes (OR typically >1.1, *p* < 0.001). This unexpected relationship may reflect the reality that high-performing nurses often assume more challenging assignments, interact with more complex cases, or bear greater responsibilities that inadvertently expose them to increased conflict situations, thereby requiring specialized protective strategies tailored to their unique professional circumstances.

### Implications for healthcare management and policy

This study reveals multidimensional factors influencing WPV among standardized training nurses, providing crucial evidence-based insights for hospital administrators to develop precise and effective prevention strategies. Based on the 18.37% prevalence rate and complex patterns of risk and protective factors, we propose the following systematic policy recommendations.

### Establishing multi-tiered psychological health support systems

Given psychological resilience as the strongest protective factor (OR = 0.837), hospitals should establish comprehensive mental health promotion mechanisms. First, implement routine psychological resilience training programs incorporating cognitive behavioral therapy, mindfulness-based stress reduction techniques, and stress management skills training to systematically enhance standardized training nurses’ psychological coping capacity. Second, establish professional Employee Assistance Programs with dedicated psychological counselors to provide timely psychological intervention and continuous support for standardized training nurses experiencing WPV or high-stress work environments. Additionally, create peer support networks by training senior nurses as mental health peer counselors, forming horizontal support systems.

### Optimizing human resource allocation and workload management

The finding that caring for more than 12 patients significantly increases violence risk (OR = 1.359) directly points to the critical role of human resource allocation. Hospital management should establish scientific nurse-to-patient ratio standards, ensuring dayshift standardized training nurses’ patient loads remain within reasonable limits. Implement dynamic bed management systems that adjust standardized training nurse allocation based on patient acuity levels and nursing care intensity requirements. Simultaneously, improve flexible scheduling systems by increasing standardized training nursing staff during peak periods to prevent service quality deterioration and conflict risks associated with excessive workloads.

### Strengthening occupational safety protection and emergency response mechanisms

Occupational exposure as a significant risk factor (OR = 1.386) underscores the importance of safety protection. Establish comprehensive occupational safety protection systems including standardized personal protective equipment allocation, standardized safety operation procedure training, regular safety risk assessments, and timely occupational exposure management mechanisms. Additionally, create violence incident emergency response systems encompassing rapid alarm mechanisms, on-site safety assurance measures, post-incident psychological crisis intervention, and legal support services to ensure nurses receive timely and effective protection when facing violent threats.

### Building cultural inclusivity and professional identity promotion mechanisms

Religious beliefs as a risk factor (OR = 1.562) reflects the importance of cultural diversity management. Hospitals should develop inclusive cultural policies and establish multicultural sensitivity training programs to enhance staff cultural understanding and communication skills. Based on professional identity as a protective factor (OR = 0.845), establish systematic professional development support plans including career planning guidance, continuing education opportunities, professional achievement recognition mechanisms, and promotion pathway optimization to strengthen nurses’ professional pride and sense of belonging.

### Improving complaint handling and conflict management systems

The finding that personal medical complaint experiences significantly increase violence risk (OR = 2.098) highlights the critical role of complaint handling mechanisms. Establish fair and transparent complaint handling procedures ensuring nurses receive adequate procedural protection and psychological support during complaint processes. Implement preventive conflict management mechanisms through patient satisfaction monitoring, early warning systems, and proactive communication interventions to effectively resolve conflicts before escalation.

### Establishing violence event monitoring and data-driven continuous improvement mechanisms

Based on witnessing colleagues’ violence as the strongest risk factor (OR = 38.490), establish hospital-wide violence event monitoring systems enabling real-time violence reporting, data analysis, and trend prediction. Through regular violence risk assessments and hotspot area identification, implement targeted preventive intervention measures. Additionally, establish data-driven continuous improvement mechanisms that regularly evaluate prevention strategy effectiveness and adjust management measures based on actual outcomes.

Implementation of these recommendations requires firm commitment from hospital senior management and interdepartmental collaboration. Through systematic policy development, resource investment, and implementation supervision, hospitals can construct safe, harmonious, and supportive work environments that effectively reduce nurses’ workplace violence risk while enhancing nursing service quality and employee wellbeing.

#### Strengths and limitations

This study’s strengths include its large, multicenter design, which enhances the generalizability of its findings. It comprehensively investigated a wide range of multifactorial determinants and analyzed WPV subtypes. The use of online data collection facilitated a high response rate.

However, the study has several limitations. First, its cross-sectional design precludes the establishment of causal relationships. Longitudinal studies are needed to confirm these associations. Second, the reliance on self-report data may be subject to recall and social desirability biases. Finally, our recruitment strategy relied on convenience sampling through online platforms such as WeChat and QR codes. This method may have introduced selection bias, as participants who chose to respond might be more technologically proficient or differ in other unmeasured characteristics from the broader population of standardized training nurses.

## Conclusion

The prevalence of WPV among nurses in standardized training in Sichuan Province, China, was lower than in many other reports, but it remains a significant concern. WPV was significantly associated with multiple factors. Key risk factors included holding religious beliefs, prior occupational exposures, high patient workload, personal history of medical complaints, and paradoxically, higher adaptive performance. Protective factors included being female, living with parents, positive career prospects, and strong job-related social support, psychological resilience, and professional identity. Therefore, comprehensive strategies aimed at improving work environments and enhancing the mental health of nurses are essential to effectively reduce the prevalence of WPV.

## Data Availability

The raw data supporting the conclusions of this article will be made available by the authors, without undue reservation.
