# Supplementary material for: Factors influencing workplace violence among Chinese nurses: a multicenter cross-sectional study
Source: Front Public Health. 2025 Dec 15;13:1697768. doi: 10.3389/fpubh.2025.1697768 (PMC12746661; doi:10.3389/fpubh.2025.1697768)
Supplement: Supplementary file 1 [file Data_Sheet_1.docx]

[Supplementary Material 1 Questionnaire on Workplace Violence Against Standardised Training Nurses 2](#_Toc26823)

[Supplementary Table1: Summary Statistics of nurses with and without experience of self-involving WPV 32](#_Toc10688)

[Supplementary Table 2 Variable assignment 35](#_Toc10347)

[Supplementary Table 3. The protective and risk factors of self-involving physical assault by ordered logit model. 36](#_Toc1620)

[Supplementary Table 4. The protective and risk factors of self-involving emotional abuse by ordered logit model. 39](#_Toc17105)

[Supplementary Table 5. The protective and risk factors of self-involving threatening by ordered logit model. 41](#_Toc7907)

[Supplementary Table 6. The protective and risk factors of self-involving verbal assault by ordered logit model. 44](#_Toc22910)

[Supplementary Table 7. The protective and risk factors of self-involving sexual assault by ordered logit model. 46](#_Toc29417)

**Supplementary Material 1 Questionnaire on Workplace Violence Against Standardized Training Nurses**

Dear Volunteer Nurses,

Greetings. I am a postgraduate student at West China Hospital of Sichuan University, currently conducting a research study on workplace violence among nurses undergoing standardized training in Sichuan Province.

This survey is conducted anonymously, and all data collected will be used solely for research purposes. We greatly appreciate your participation. There are no right or wrong answers to the questions; please select the option that best reflects your personal experience.

Thank you very much for taking the time to complete this questionnaire.

**Informed Consent Form**

Please read this consent form. If you have any questions, feel free to ask the researcher, who will answer them accordingly.

This study concerns workplace violence among nurses undergoing standardised training in Sichuan Province and has been approved by the Ethics Committee of West China Hospital (Approval No: 2023822). You are welcome to take part in this research if you wish. Participation is entirely voluntary, and you may withdraw at any time, even after giving consent. Your responses will be kept strictly confidential and will not be disclosed to your employer.

To help identify factors associated with workplace violence, you will be asked to complete several questionnaires. This will take no more than 30 minutes, and all responses will be anonymous.

Please indicate whether you agree to participate in the study:

1. Yes, I agree to participate

Participant’s signature (a nickname is acceptable)

1. No, I do not agree to participate
2. **Have you personally experienced workplace violence in a healthcare setting?**
3. Yes (Skip to 2)
4. No
5. **Nature of Workplace Violence Experienced**
6. Physical assault (e.g., being spat at, bitten, hit, pushed, kicked, grabbed, etc.)
7. Emotional abuse (e.g., verbal insults, humiliation, aggressive shouting, etc.)
8. Threats (including verbal, written, physical gestures, or threats with weapons)
9. Harassment of a verbal nature from the opposite sex
10. Harassment of a physical nature from the opposite sex (unwanted touching or physical advances)
11. **Have you witnessed colleagues experiencing workplace violence in a healthcare setting?**
12. Yes (Skip to 4)
13. No

**4.Nature of Workplace Violence Witnessed**

1. Witnessed others experiencing physical assault (e.g., spat at, bitten, hit, pushed, kicked, grabbed, etc.)
2. Witnessed others experiencing emotional abuse (e.g., verbal insults, humiliation, aggressive shouting, etc.)
3. Witnessed others being threatened (including verbal, written, physical gestures, or threats with weapons)
4. Witnessed others experiencing verbal harassment from the opposite sex
5. Witnessed others experiencing physical harassment from the opposite sex (unwanted touching or physical advances)

**Part I Demographic characteristics**

1. **What is your gender?**
2. Male
3. Female
4. **What is your age? (years old)**

**___________________**

1. **What is your region of household registration?**
2. Urban
3. Rural
4. **What is your educational level?**
5. Vocational high school
6. Two-year college
7. Bachelor’s degree or above
8. **How much is your family income per month (CNY)?**

**___________________**

1. **How much is your own income per month (CNY)?**

**___________________**

1. **What is your marital status?**
2. Single
3. Married
4. Others
5. **Do you have religious beliefs?**
6. **Yes**
7. **No**
8. **Number of household members**

**___________________**

1. **Do you live with your parent(s)?**
2. Yes
3. No
4. **Do you have a 'left-behind' experience (defined as: Before the age of 16, did you remain in your hometown for more than three months while either one or both of your parents migrated for work)?**
5. Yes
6. No

**PartⅡ Working conditions**

1. **What is the level of your training hospital?**
2. Tertiary Ⅲ
3. Non-tertiary Ⅲ

**2.Have you ever experienced occupational exposure (e.g., needlestick injuries or radiation exposure)?**

1. Yes
2. No

3.**What is your working length ( hours/week)?**

1. Within 40 hours
2. 41 to 55 hours
3. 56 to 70 hours
4. More than 70 hours

**4.Day shift**

Number of day shifts per week: _________

Average length per day shift: _________ hours

1. **Night shift**

Number of night shifts per week: _________

Average length per night shift: _________ hours

1. **What is the average number of patients assigned to you during a day shift?**

**___________________**

1. **Number of patient deaths after cared in the past six months: ______**
2. **Daily commute time to work: ______ hours**

**9.Have you ever been the subject of a medical complaint?**

1. Yes
2. No

**10.Have you ever witnessed a colleague be subjected to a medical complaint?**

1. Yes
2. No

**11.Job Satisfaction: To what extent do you feel a balance between your effort and return?**

1. Very unsatisfied
2. Unsatisfied
3. Neutral
4. Satisfied
5. Very satisfied

**12.How do you perceive the prospects for your future professional status?**

1. Below current level
2. Unchanged
3. Higher than current level

**13Job content questionnaire (JCQ)**

**Section 1:Job demands**

**Q1:My job requires me to work very fast.**

1. Strongly disagree
2. Disagree
3. Somewhat agree
4. Agree
5. Strongly agree

**Q2:My job requires me to work very hard.**

1. Strongly disagree
2. Disagree
3. Somewhat agree
4. Agree
5. Strongly agree

**Q3：There is no requirement to work overtime.**

1. Strongly disagree
2. Disagree
3. Somewhat agree
4. Agree
5. Strongly agree

**Q4：There is enough time to complete work tasks.**

1. Strongly disagree
2. Disagree
3. Somewhat agree
4. Agree
5. Strongly agree

**Q5：There is no need to do contradictory things based on conflicting requests from others.**

1. Strongly disagree
2. Disagree
3. Somewhat agree
4. Agree
5. Strongly agree

**Section 2:Job control**

**Q6:The job requires learning new things.**

1. Strongly disagree
2. Disagree
3. Somewhat agree
4. Agree
5. Strongly agree

**Q7:The work is highly repetitive.**

1. Strongly disagree
2. Disagree
3. Somewhat agree
4. Agree
5. Strongly agree

**Q8:The job requires creativity.**

1. Strongly disagree
2. Disagree
3. Somewhat agree
4. Agree
5. Strongly agree

**Q9:In the job, one can make many decisions independently.**

1. Strongly disagree
2. Disagree
3. Somewhat agree
4. Agree
5. Strongly agree

**Q10:The job demands high technical skills.**

1. Strongly disagree
2. Disagree
3. Somewhat agree
4. Agree
5. Strongly agree

**Q11:There is almost no authority to decide how to carry out the work.**

1. Strongly disagree
2. Disagree
3. Somewhat agree
4. Agree
5. Strongly agree

**Q12：In the job, one can do a variety of different tasks.**

1. Strongly disagree
2. Disagree
3. Somewhat agree
4. Agree
5. Strongly agree

**Q13：One’s opinions have influence over things that happen at work.**

1. Strongly disagree
2. Disagree
3. Somewhat agree
4. Agree
5. Strongly agree

**Q14：There are opportunities to use one’s special abilities in the job.**

1. Strongly disagree
2. Disagree
3. Somewhat agree
4. Agree
5. Strongly agree

**Section 3:Social support**

**Q15：Supervisors show concern for the welfare of subordinates.**

1. Strongly disagree
2. Disagree
3. Somewhat agree
4. Agree
5. Strongly agree

**Q16:Supervisors listen to employees’ opinions.**

1. Strongly disagree
2. Disagree
3. Somewhat agree
4. Agree
5. Strongly agree

**Q17:Supervisors help subordinates with their work.**

1. Strongly disagree
2. Disagree
3. Somewhat agree
4. Agree
5. Strongly agree

**Q18:Supervisors are well-organized and enable subordinates to work cohesively.**

1. Strongly disagree
2. Disagree
3. Somewhat agree
4. Agree
5. Strongly agree

**Q19：Colleagues are competent in their work.**

1. Strongly disagree
2. Disagree
3. Somewhat agree
4. Agree
5. Strongly agree

**Q20：Colleagues show concern for me.**

1. Strongly disagree
2. Disagree
3. Somewhat agree
4. Agree
5. Strongly agree

**Q21：Colleagues are friendly.**

1. Strongly disagree
2. Disagree
3. Somewhat agree
4. Agree
5. Strongly agree

**Q22：Colleagues provide help in the workplace.**

1. Strongly disagree
2. Disagree
3. Somewhat agree
4. Agree
5. Strongly agree

**Part Ⅲ Lifestyles**

1. **Sleep**

**Q1:How many hours of sleep do you get on average per day?**

**___________________**

**Q2:What is the quality of your sleep like?**

1. good
2. normal
3. Bad

**Q3:Do you nap at lunchtime?**

1. Yes
2. No

**2.Do you consume alcohol?**(Alcohol consumption is defined as: drinking more than 50 ml of spirits or wine, or 200 ml of beer per occasion, at least twice weekly, consistently for over 6 months. Occasional minimal consumption during holidays is not considered alcohol consumption.)

1. Yes
2. No

**3.Do you smoke?**(Definition of smoking: Smoking at least one cigarette per day for a consecutive period of six months or more, or having smoked a cumulative total of 150 cigarettes or more.)

1. Yes
2. No
3. **Exercise**

**Q1:Do you have exercise habit?**(Exercise is defined as: a planned and repetitive physical activity undertaken to improve or maintain physical health, such as running, yoga, swimming, hiking, etc.)

1. Yes
2. No

**Q2:How often do you exercise per week?**

1. 1-2 times
2. 3-4 times
3. >5 times

**Q3：Duration of exercise per session: ______ minutes?**

**Part Ⅳ：Mental and physical health**

1. **Chinese version of the Resilience Scale-14 (RS-14)**

**Q1: I usually manage one way or another.**

1. Never
2. Rarely
3. Sometimes
4. Often
5. Always

**Q2: I feel proud that I have accomplished things in life.**

1. Never
2. Rarely
3. Sometimes
4. Often
5. Always

**Q3: I usually take things in stride.**

1. Never
2. Rarely
3. Sometimes
4. Often
5. Always

**Q4: I am friends with myself.**

1. Never
2. Rarely
3. Sometimes
4. Often
5. Always

**Q5: I feel that I can handle many things at a time.**

1. Never
2. Rarely
3. Sometimes
4. Often
5. Always

**Q6: I am determined.**

1. Never
2. Rarely
3. Sometimes
4. Often
5. Always

**Q7: I can get through difficult times, because I have experienced difficulty before.**

1. Never
2. Rarely
3. Sometimes
4. Often
5. Always

**Q8: I have self-discipline.**

1. Never
2. Rarely
3. Sometimes
4. Often
5. Always

**Q9: I keep interested in things.**

1. Never
2. Rarely
3. Sometimes
4. Often
5. Always

**Q10:I can usually find something to laugh about.**

1. Never
2. Rarely
3. Sometimes
4. Often
5. Always

**Q11:My belief in myself gets me through hard times.**

1. Never
2. Rarely
3. Sometimes
4. Often
5. Always

**Q12: In an emergency, I am someone people can generally rely on.**

1. Never
2. Rarely
3. Sometimes
4. Often
5. Always

**Q13: My life has meaning.**

1. Never
2. Rarely
3. Sometimes
4. Often
5. Always

**Q14:When I am in a difficult situation, I can usually find my way out of it.**

1. Never
2. Rarely
3. Sometimes
4. Often
5. Always
6. **Adaptive Performance Questionnaire,APO**

**Section 1:Stress and emergency handling**

**Q1:Maintain composure when work schedules are excessively tight.**

1. Strongly disagree
2. Somewhat disagree
3. Neutral
4. Somewhat agree
5. Strongly agree

**Q2:Remain calm under excessive work pressure.**

1. Strongly disagree
2. Somewhat disagree
3. Neutral
4. Somewhat agree
5. Strongly agree

**Q3:Maintain emotional control when dealing with emergencies.**

1. Strongly disagree
2. Somewhat disagree
3. Neutral
4. Somewhat agree
5. Strongly agree

**Q4:Generally think clearly and prioritise effectively when handling emergencies.**

1. Strongly disagree
2. Somewhat disagree
3. Neutral
4. Somewhat agree
5. Strongly agree

**Q5:Handle emergency situations objectively.**

1. Strongly disagree
2. Somewhat disagree
3. Neutral
4. Somewhat agree
5. Strongly agree

**Section 2:Interpersonal and cultural adaptation**

**Q6:Work effectively with people of diverse personalities.**

1. Strongly disagree
2. Somewhat disagree
3. Neutral
4. Somewhat agree
5. Strongly agree

**Q7:Integrate different values, customs and cultures.**

1. Strongly disagree
2. Somewhat disagree
3. Neutral
4. Somewhat agree
5. Strongly agree

**Q8:Maintain good relationships with people from different cultures.**

1. Strongly disagree
2. Somewhat disagree
3. Neutral
4. Somewhat agree
5. Strongly agree

**Q9:Adapt one's behaviour to accommodate other cultures and customs.**

1. Strongly disagree
2. Somewhat disagree
3. Neutral
4. Somewhat agree
5. Strongly agree

**Q10:Adjust one's behaviour when necessary.**

1. Strongly disagree
2. Somewhat disagree
3. Neutral
4. Somewhat agree
5. Strongly agree

**Q11:Proactively learn about the working atmosphere and needs of other departments.**

1. Strongly disagree
2. Somewhat disagree
3. Neutral
4. Somewhat agree
5. Strongly agree

**Q12:Understand the organisational climate, direction and values of the company.**

1. Strongly disagree
2. Somewhat disagree
3. Neutral
4. Somewhat agree
5. Strongly agree

**Q13:Easily understand the meaning of behaviours in other cultures and adapt accordingly.**

1. Strongly disagree
2. Somewhat disagree
3. Neutral
4. Somewhat agree
5. Strongly agree

**3.Professional identity**

**Q1:Nursing work gives me a sense of value.**

1. Strongly disagree
2. Somewhat disagree
3. Neutral
4. Somewhat agree
5. Strongly agree

**Q2:I feel confident in approaching and interacting with anyone I meet.**

1. Strongly disagree
2. Somewhat disagree
3. Neutral
4. Somewhat agree
5. Strongly agree

**Q3:Colleagues’ understanding and support give me a sense of fulfilment.**

1. Strongly disagree
2. Somewhat disagree
3. Neutral
4. Somewhat agree
5. Strongly agree

**Q4:I firmly believe that humans have subjective choice and agency, enabling them not only to adapt to but also to create and optimise their professional environment.**

1. Strongly disagree
2. Somewhat disagree
3. Neutral
4. Somewhat agree
5. Strongly agree

**Q5:I believe that career choice involves both inevitability and serendipity; one must embrace it to achieve meaningful outcomes.**

1. Strongly disagree
2. Somewhat disagree
3. Neutral
4. Somewhat agree
5. Strongly agree

**Q6:Nursing aligns well with my interests and personality.**

1. Strongly disagree
2. Somewhat disagree
3. Neutral
4. Somewhat agree
5. Strongly agree

**Q7:The nursing profession allows me to more easily earn respect from the broader public.**

1. Strongly disagree
2. Somewhat disagree
3. Neutral
4. Somewhat agree
5. Strongly agree

**Q8:I enjoy communicating with people and actively seek out social contact.**

1. Strongly disagree
2. Somewhat disagree
3. Neutral
4. Somewhat agree
5. Strongly agree

**Q9:Recognition from doctors and managers gives me a sense of fulfilment.**

1. Strongly disagree
2. Somewhat disagree
3. Neutral
4. Somewhat agree
5. Strongly agree

**4.Social Support Rating Scale (SSRS)**

**Q1: How many close friends do you have whom you can rely on for support and help?**

1. None
2. 1-2
3. 3-5
4. 6 or more

**Q2: Which of the following best describes your living situation over the past year? (Select only one option)**

1. Lived away from family and resided alone
2. Frequently changed residence and mostly lived with strangers
3. Lived with classmates, colleagues, or friends
4. Lived with family

**Q3: Which of the following best describes your relationship with your neighbours? (Select only one option)**

1. We do not show concern for each other and are merely nodding acquaintances
2. Might show occasional concern if difficulties arise
3. Some neighbours show genuine concern for me
4. Most neighbours show genuine concern for me

**Q4: Which of the following best describes your relationship with your colleagues? (Select only one option)**

1. We do not show concern for each other and are merely nodding acquaintances
2. Might show occasional concern if difficulties arise
3. Some colleagues show genuine concern for me
4. Most colleagues show genuine concern for me

**Q5: Support and care received from family members (Tick "√" in the appropriate box)**

|  | **No support** | **Minimal support** | **Moderate support** | **Full support** |
| --- | --- | --- | --- | --- |
| **A.Spouse/Partner** |  |  |  |  |
| **B.Parents** |  |  |  |  |
| **C.Children** |  |  |  |  |
| **D.Siblings** |  |  |  |  |
| **E.Other relatives (e.g., in-laws)** |  |  |  |  |

**Q6: In the past, which sources have provided you with financial support or practical help during critical or emergency situations?**

1. No sources
2. The following sources (multiple selections allowed):

A. Spouse; B. Other family members; C. Friends; D. Relatives; E. Colleagues; F. Work unit/Employer; G. Official or semi-official organisations (e.g., political parties, leagues, trade unions); H. Non-official organisations (e.g., religious or social groups); I. Other (please specify)

**Q7: In the past, which sources have provided you with comfort and concern during critical or emergency situations?**

1. No sources
2. The following sources (multiple selections allowed):

A. Spouse; B. Other family members; C. Friends; D. Relatives; E. Colleagues; F. Work unit/Employer; G. Official or semi-official organisations (e.g., political parties, leagues, trade unions); H. Non-official organisations (e.g., religious or social groups); I. Other (please specify)

**Q8: How do you typically confide in others when encountering personal troubles? (select only one option)**

1. Never confide in anyone
2. Only confide in 1-2 people with whom I have a very close relationship
3. Share only if friends actively inquire
4. Actively discuss my troubles to seek support and understanding

**Q9: How do you seek help when facing difficulties? (select only one option)**

1. Rely solely on myself and do not accept help from others
2. Rarely ask others for help
3. Occasionally ask others for help
4. Often seek help from family, friends, or organisations when encountering difficulties

**Q10: Regarding activities organised by groups (e.g., political leagues, religious organisations, trade unions, student unions, etc.), you: (select only one option)**

1. Never participate
2. Occasionally participate
3. Often participate
4. Actively participate and engage

**Q1：Being required by others to perform tasks outside of my job scope, e.g., running personal errands.**

1. Very Rarely
2. Rarely
3. Occasionally
4. Frequently
5. Very Frequently

**Q2:Being subjected to closer supervision/monitoring than other colleagues.**

1. Very Rarely
2. Rarely
3. Occasionally
4. Frequently
5. Very Frequently

**Q3:I have been excessively teased, humiliated, or offended by colleagues or superiors.**

1. Very Rarely
2. Rarely
3. Occasionally
4. Frequently
5. Very Frequently

**Q4:Having to work much harder than other colleagues in order to get my work done.**

1. Very Rarely
2. Rarely
3. Occasionally
4. Frequently
5. Very Frequently

**Q5:My supervisor belittles or undervalues my work and efforts.**

1. Very Rarely
2. Rarely
3. Occasionally
4. Frequently
5. Very Frequently

**Q6:Being unfairly humiliated at work.**

1. Very Rarely
2. Rarely
3. Occasionally
4. Frequently
5. Very Frequently

**6.Have you been diagnosed with any chronic diseases in the past year?**

(Chronic diseases: Refers to non-communicable conditions characterized by long-term accumulation and persistent health impairment. Examples include cardiovascular and cerebrovascular diseases, cancers, diabetes mellitus, chronic respiratory diseases, etc.)

1. Yes (Skip to 7)
2. No

**7.Which chronic disease(s) have you been diagnosed with?**

1. Hypertension
2. Asthma
3. Diabetes
4. Others

**Supplementary Table1: Summary Statistics of nurses with and without experience of self-involving WPV**

|  | Total (n=7231) | Self-involving WPV | No Self-involving WPV | t or Pearson chi2 | *p* |
| --- | --- | --- | --- | --- | --- |
|  |  | (n=1328) | (n=5903) |  |  |
| ***Demographic characteristics*** |  |  |  |  |  |
| Gender |  |  |  |  |  |
| male | 516 | 134 (25.97%) | 382 (74.03%) | 21.427 | < 0.001 |
| female | 6,715 | 1,194 (17.78%) | 5,521 (82.22%) |  |  |
| Age | 23.02 (1.61) | 23.20 (1.61) | 22.98 (1.61) | -4.378 | < 0.001 |
| Region |  |  |  |  |  |
| urban | 2,017 | 420 (20.82%) | 1,597 (79.18%) | 11.269 | 0.001 |
| rural | 5,214 | 908 (17.41%) | 4,306 (82.59%) |  |  |
| Education |  |  |  |  |  |
| vocational high school | 179 | 38 (21.23%) | 141 (78.77%) | 17.68 | < 0.001 |
| two-year college | 5,258 | 904 (17.19%) | 4,354 (82.81%) |  |  |
| postgraduate | 1,794 | 386 (21.52%) | 1,408 (78.48%) |  |  |
| Marital status |  |  |  |  |  |
| single | 6,621 | 1,223 (18.47%) | 5,398 (81.53%) | 0.616 | 0.795 |
| married | 579 | 100 (17.27%) | 479 (82.73%) |  |  |
| others | 31 | 5 (16.13%) | 26 (83.87%) |  |  |
| Religious belief |  |  |  |  |  |
| yes | 411 | 110 (26.76%) | 301 (73.24%) | 20.502 | < 0.001 |
| no | 6,820 | 1,218 (17.86%) | 5,602 (82.14%) |  |  |
| no | 7,018 | 1,264 (18.01%) | 5,754 (81.99%) |  |  |
| Number of household members | 3.86 (1.26) | 3.72 (1.27) | 3.89 (1.25) | 4.421 | < 0.001 |
| Living with parents |  |  |  |  |  |
| yes | 3,700 | 599 (16.19%) | 3,101 (83.81%) | 23.934 | < 0.001 |
| no | 3,531 | 729 (20.65%) | 2,802 (79.35%) |  |  |
| Experience of being left behind |  |  |  |  |  |
| yes | 3,631 | 727 (20.02%) | 2,904 (79.98%) | 13.351 | < 0.001 |
| no | 3,600 | 601 (16.69%) | 2,999 (83.31%) |  |  |
| ***Working Conditions*** |  |  |  |  |  |
| Hospital level |  |  |  |  |  |
| tertiary III | 6,213 | 1,148 (18.48%) | 5,065 (81.52%) | 0.369 | 0.543 |
| non-tertiary III | 1,018 | 180 (17.68%) | 838 (82.32%) |  |  |
| Occupational exposure |  |  |  |  |  |
| yes | 3,515 | 863 (24.55%) | 2,652 (75.45%) | 174.61 | < 0.001 |
| no | 3,716 | 465 (12.51%) | 3,251 (87.49%) |  |  |
| Working length (hours/week) |  |  |  |  |  |
| within 40 hours | 2,085 | 302 (14.48%) | 1,783 (85.52%) | 36.538 | < 0.001 |
| 41 to 55 hours | 4,195 | 814 (19.40%) | 3,381 (80.60%) |  |  |
| 56 to 70 hours | 754 | 160 (21.22%) | 594 (78.78%) |  |  |
| more than 70 hours | 197 | 52 (26.40%) | 145 (73.60%) |  |  |
| Income (family/monthly) | 3638.74 (3799.42) | 3849.46 (4116.80) | 3591.34 (3723.02) | -2.238 | 0.025 |
| Income (self/monthly) | 2614.63 (1142.76) | 2750.98 (1193.93) | 2583.96 (1128.77) | -4.82 | < 0.0001 |
| Day shift frequency per week |  |  |  |  |  |
| Day shift length (hours) | 8.20 (0.75) | 8.29 (0.77) | 8.18 (0.74) | -4.578 | < 0.0001 |
| Night shift frequency per month |  |  |  |  |  |
| Night shift length | 8.36 (1.28) | 8.48 (1.26) | 8.33 (1.28) | -3.719 | 0.0002 |
| Caring patients (no.) | 9.37 (6.01) | 10.30 (6.29) | 9.17 (5.92) | -6.209 | < 0.0001 |
| Patients’ death (no.) | 1.15 (2.72) | 1.46 (3.13) | 1.08 (2.61) | -4.59 | < 0.0001 |
| Daily commuting time to work (hours) | 0.93 (0.56) | 0.97 (0.60) | 0.92 (0.55) | -3.231 | 0.0012 |
| Own medical complaints |  |  |  |  |  |
| yes | 214 | 104 (48.60%) | 110 (51.40%) | 134.444 | < 0.0001 |
| no | 7,017 | 1,224 (17.44%) | 5,793 (82.56%) |  |  |
| Medical complaints of colleagues |  |  |  |  |  |
| yes | 3,233 | 867 (26.82%) | 2,366 (73.18%) | 278.605 | < 0.0001 |
| no | 3,998 | 461 (11.53%) | 3,537 (88.47%) |  |  |
| Feel a balance between effort and return |  |  |  |  |  |
| very unsatisfied | 31 | 9 (29.03%) | 22 (70.97%) | 176.149 | < 0.0001 |
| unsatisfied | 50 | 26 (52.00%) | 24 (48.00%) |  |  |
| normal | 2,420 | 605 (25.00%) | 1,815 (75.00%) |  |  |
| satisfied | 4,060 | 631 (15.54%) | 3,429 (84.46%) |  |  |
| very satisfied | 670 | 57 (8.51%) | 613 (91.49%) |  |  |
| Career prospects |  |  |  |  |  |
| below current level | 492 | 163 (33.13%) | 329 (66.87%) | 116.533 | < 0.0001 |
| unchanged | 2,009 | 439 (21.85%) | 1,570 (78.15%) |  |  |
| higher than current level | 4,730 | 726 (15.35%) | 4,004 (84.65%) |  |  |
| Job demands | 18.05 (2.83) | 16.99 (2.83) | 18.29 (2.78) | 15.285 | < 0.0001 |
| Job control | 32.15 (4.14) | 31.03 (4.08) | 32.40 (4.11) | 11.009 | < 0.0001 |
| Job social support | 29.62 (5.13) | 27.73 (5.38) | 30.05 (4.98) | 15.084 | < 0.0001 |
| Degree of job stress | 1.01 (0.12) | 0.99 (0.13) | 1.02 (0.12) | 7.921 | < 0.0001 |
| ***Lifestyle*** |  |  |  |  |  |
| Length of sleep (hours per day) | 6.92 (0.86) | 6.73 (0.85) | 6.96 (0.85) | 9.228 | < 0.0001 |
| Sleep quality |  |  |  |  |  |
| good | 1,598 | 198 (12.39%) | 1,400 (87.61%) | 114.186 | < 0.0001 |
| normal | 4,463 | 800 (17.93%) | 3,663 (82.07%) |  |  |
| bad | 1,170 | 330 (28.21%) | 840 (71.79%) |  |  |
| Nap at lunchtime |  |  |  |  |  |
| yes | 4,992 | 878 (17.59%) | 4,114 (82.41%) | 6.496 | 0.011 |
| no | 2,239 | 450 (20.10%) | 1,789 (79.90%) |  |  |
| Alcohol consumption |  |  |  |  |  |
| yes | 154 | 43 (27.92%) | 111 (72.08%) | 9.585 | 0.002 |
| no | 7,077 | 1,285 (18.16%) | 5,792 (81.84%) |  |  |
| Smoking |  |  |  |  |  |
| yes | 122 | 36 (29.51%) | 86 (70.49%) | 10.277 | 0.001 |
| no | 7,109 | 1,292 (18.17%) | 5,817 (81.83%) |  |  |
| Exercise habits |  |  |  |  |  |
| yes | 2,519 | 377 (14.97%) | 2,142 (85.03%) | 29.791 | < 0.0001 |
| no | 4,712 | 951 (20.18%) | 3,761 (79.82%) |  |  |
| Exercise frequency (per week) |  |  |  |  |  |
| 1-2 times | 6,649 | 1,229 (18.48%) | 5,420 (81.52%) | 1.085 | 0.581 |
| 3-4 times | 470 | 82 (17.45%) | 388 (82.55%) |  |  |
| >5 times | 112 | 17 (15.18%) | 95 (84.82%) |  |  |
| Exercise length (min per time) | 16.44 (27.88) | 14.59 (29.42) | 16.86 (27.51) | 2.685 | 0.0073 |
| ***Mental and physical health*** |  |  |  |  |  |
| Resilience total score | 49.49 (8.32) | 47.00 (8.35) | 50.04 (8.22) | 12.178 | < 0.0001 |
| Resilience (factor 1) | 34.84 (6.09) | 33.04 (6.10) | 35.24 (6.01) | 12.017 | < 0.0001 |
| Resilience (factor 2) | 14.65 (2.49) | 13.96 (2.57) | 14.81 (2.45) | 11.303 | < 0.0001 |
| Adaptive performance | 226.84(26.14) | 222.47 (26.02) | 227.82 (26.07) | 6.76 | < 0.0001 |
| Social support | 8.13 (1.86) | 7.76 (1.82) | 8.21 (1.86) | 7.986 | < 0.0001 |
| Professional identity | 32.93 (5.71) | 30.48 (5.84) | 33.48 (5.53) | 17.635 | < 0.0001 |
| Bullying | 12.93 (4.76) | 13.92 (5.00) | 12.71 (4.68) | -8.409 | < 0.0001 |
| Chronic disease |  |  |  |  |  |
| yes | 232 | 68 (29.31%) | 164 (70.69%) | 19.152 | < 0.001 |
| no | 6,999 | 1,260 (18.00%) | 5,739 (82.99%) |  |  |
| Hypertension |  |  |  |  |  |
| yes | 10 | 2 (20.00%) | 8 (80.00%) | 0.0178 | 0.894 |
| no | 7221 | 1,326 (18.36%) | 5,895 (81.64%) |  |  |
| Asthma |  |  |  |  |  |
| yes | 11 | 2 (18.18%) | 9 (81.82%) | 0.0002 | 0.987 |
| no | 7,220 | 1,326 (18.37%) | 5,894 (81.63%) |  |  |
| Diabetes |  |  |  |  |  |
| yes | 11 | 2 (18.18%) | 9 (81.82%) | 0.0002 | 0.987 |
| no | 7,220 | 1,326 (18.37%) | 5,894 (81.63%) |  |  |
| Others |  |  |  |  |  |
| yes | 213 | 64 (30.05%) | 149 (69.95%) | 19.975 | < 0.001 |
| no | 7,018 | 1,264 (18.01%) | 5,754 (81.99%) |  |  |

Note: For continuous variables, data were presented as mean and SD if normally distributed, if not, data were presented as median with min and max values. The student t test was used to compare the between groups difference if normally distributed, if not, non-parametric test was applied.

**Supplementary Table 2** Variable assignment

| **Variables** | **Assignment** |
| --- | --- |
| ***Demographic characteristics*** |  |
| Gender | male=0; female=1 |
| Age | actual numerical value |
| Region of hospital | rural=0; urban=1 |
| Education level | vocational high school=0; two-year college=1; postgraduate=2 |
| Marital status | single=0; married=1; others=2 |
| Religious belief | no=1; yes=1 |
| Number of household members | 0 person=0; one person=1; two persons=2; ＞two persons=3 |
| Living with parents | no=0; yes=1 |
| Experience of being left behind | no=0; yes=1 |
| ***Working Conditions*** |  |
| Hospital level | non-tertiary III=0; tertiary III=1 |
| Occupational exposure | no=0; yes=1 |
| Working length (hours/week) | within 40 hours=0; 41 to 55 hours=1; 56 to 70 hours=2; more than 70 hours=3 |
| Own income per month | less than 1000 RMB=0; below China’s national average disposable income=1; above China’s national average disposable income |
| Day shift frequency per week | < 15 days=0; 15 to 22 days=1; > 22 days=2 |
| Day shift length (hours) | actual numerical value |
| Night shift frequency per month | < 2 times=0; 2 to 6 times=1; > 6 times=2 |
| Night shift length | actual numerical value |
| Caring patients (no.) | < 4=0; 5-8=1; 9-12=2; ＞12=4 |
| Patients’ death (no.) | 0 patient death=0; one=1; > one=2 |
| Daily commuting time to work (hours) | actual numerical value |
| Own medical complaints | no=0; yes=1 |
| Medical complaints of colleagues | no=0; yes=1 |
| Feel a balance between effort and return | very unsatisfied=0; unsatisfied=1; neutral=2; satisfied=3; very satisfied=4 |
| Career prospects | Below current level=0; unchanged=1; higher than current level=2 |
| Job demands | actual numerical value |
| Job control | actual numerical value |
| Job social support | actual numerical value |
| Degree of job stress | actual numerical value |
| ***Lifestyles*** |  |
| Nap at lunchtime | no=0; yes=1 |
| Alcohol consumption | no=0; yes=1 |
| Smoking | no=0; yes=1 |
| Exercise habits | no=0; yes=1 |
| Exercise frequency (per week) | 1-2 times=0; 3-4 times=1 |
| Exercise duration (min/time) | actual numerical value |
| ***Mental and physical health*** |  |
| Resilience total score | actual numerical value |
| Adaptive performance | actual numerical value |
| Social support | actual numerical value |
| Professional identity | actual numerical value |
| Bullying | actual numerical value |
| ***Workplace Violence Exposure*** |  |
| Witness of colleagues’ WPV | no=0; yes=1; Witness of colleagues’ physical assault=2; Witness of colleagues’ emotional abuse=3; Witness of colleagues’ threatening=4; Witness of colleagues’ verbal assault=5; Witness of colleagues’ sexual assault=6 |

**Supplementary Table 3. The protective and risk factors of self-involving physical assault by ordered logit model.**

| **Variables** | β | SE | OR | 95% CI of OR | | *p*  value |
| --- | --- | --- | --- | --- | --- | --- |
|  |  |  |  | lower | upper |  |
| ***Demographic characteristics*** |  |  |  |  |  |  |
| **Gender** |  |  |  |  |  |  |
| female | 0.145 | -2.030 | **0.626*** | 0.398 | 0.985 | 0.043 |
| Age | 0.054 | 0.840 | 1.044 | 0.944 | 1.154 | 0.403 |
| Region |  |  |  |  |  |  |
| urban | 0.165 | 0.100 | 1.016 | 0.739 | 1.398 | 0.921 |
| Education |  |  |  |  |  |  |
| two-year college | 0.341 | -0.710 | 0.714 | 0.280 | 1.189 | 0.480 |
| postgraduate | 0.323 | -0.890 | 0.639 | 0.237 | 1.720 | 0.375 |
| Marital status |  |  |  |  |  |  |
| married | 0.563 | -0.560 | 0.579 | 0.086 | 3.894 | 0.574 |
| others | 0.600 | -0.480 | 0.636 | 0.100 | 4.036 | 0.631 |
| **Religious belief** |  |  |  |  |  |  |
| **yes** | 0.468 | 2.790 | **1.954**** | 1.221 | 3.126 | 0.005 |
| Number of household members |  |  |  |  |  |  |
| one person | 0.890 | 1.230 | 1.820 | 0.698 | 4.745 | 0.220 |
| two persons | 0.802 | 1.220 | 1.751 | 0.713 | 4.296 | 0.222 |
| > two persons | 0.733 | 1.080 | 1.629 | 0.674 | 3.933 | 0.278 |
| **Living with parents** |  |  |  |  |  |  |
| **yes** | 0.111 | -2.070 | **0.730*** | 0.542 | 0.983 | 0.038 |
| Experience of being left behind |  |  |  |  |  |  |
| yes | 0.158 | 0.090 | 1.015 | 0.748 | 1.377 | 0.924 |
| ***Working Conditions*** |  |  |  |  |  |  |
| Hospital level |  |  |  |  |  |  |
| tertiary III | 0.356 | 1.570 | 1.465 | 0.910 | 2.358 | 0.116 |
| Occupational exposure |  |  |  |  |  |  |
| yes | 0.148 | -0.290 | 0.957 | 0.707 | 1.295 | 0.775 |
| Working length (hours/week) |  |  |  |  |  |  |
| 41 to 55 hours | 0.162 | -0.730 | 0.873 | 0.606 | 1.257 | 0.464 |
| 56 to 70 hours | 0.278 | 0.170 | 1.045 | 0.620 | 1.762 | 0.869 |
| more than 70 hours | 0.563 | 1.120 | 1.518 | 0.734 | 3.140 | 0.261 |
| Own income per month |  |  |  |  |  |  |
| below China’s national average disposable income | 0.207 | -1.660 | 0.510 | 0.230 | 1.131 | 0.097 |
| above China’s national average disposable income | 0.282 | -0.820 | 0.726 | 0.339 | 1.555 | 0.410 |
| Day shift frequency per week |  |  |  |  |  |  |
| 15 to 22 days | 0.159 | -1.200 | 0.784 | 0.526 | 1.167 | 0.231 |
| > 22 days | 0.217 | -0.440 | 0.898 | 0.559 | 1.443 | 0.657 |
| Day shift length (hours) | 0.094 | -0.950 | 0.906 | 0.739 | 1.111 | 0.343 |
| **Night shift frequency per month** |  |  |  |  |  |  |
| **2 to 6 times** | 0.272 | 2.060 | **1.465*** | 1.018 | 2.106 | 0.040 |
| > 6 times | 0.311 | 0.890 | 1.248 | 0.766 | 2.033 | 0.373 |
| Night shift length | 0.062 | 0.680 | 1.041 | 0.926 | 1.170 | 0.499 |
| **Caring patients (no.)** |  |  |  |  |  |  |
| 5-8 | 0.300 | 1.070 | 1.284 | 0.812 | 2.029 | 0.285 |
| 9-12 | 0.252 | 0.430 | 1.103 | 0.705 | 1.725 | 0.667 |
| **>12** | 0.325 | 2.010 | **1.533*** | 1.011 | 2.323 | 0.044 |
| Patients’ death (no.) |  |  |  |  |  |  |
| one | 0.240 | -0.340 | 0.914 | 0.546 | 1.528 | 0.731 |
| > one | 0.194 | 1.270 | 1.224 | 0.896 | 1.671 | 0.204 |
| Daily commuting time to work (hours) | 0.117 | -0.900 | 0.888 | 0.686 | 1.150 | 0.367 |
| Own medical complaints |  |  |  |  |  |  |
| yes | 0.453 | 1.750 | 1.627 | 0.943 | 2.806 | 0.080 |
| Medical complaints of colleagues |  |  |  |  |  |  |
| yes | 0.129 | -1.610 | 0.762 | 0.547 | 1.061 | 0.107 |
| Feel a balance between effort and return |  |  |  |  |  |  |
| unsatisfied | 0.516 | -0.550 | 0.642 | 0.133 | 3.104 | 0.581 |
| neutral | 0.499 | -0.300 | 0.835 | 0.258 | 2.696 | 0.763 |
| satisfied | 0.636 | 0.100 | 1.062 | 0.329 | 3.435 | 0.920 |
| very satisfied | 0.552 | -0.310 | 0.806 | 0.211 | 3.084 | 0.753 |
| Career prospects |  |  |  |  |  |  |
| unchanged | 0.304 | 0.620 | 1.173 | 0.706 | 1.948 | 0.539 |
| higher than current level | 0.241 | -0.190 | 0.952 | 0.580 | 1.562 | 0.845 |
| Job demands | 0.138 | -1.470 | 0.768 | 0.540 | 1.093 | 0.143 |
| Job control | 0.096 | -1.310 | 0.866 | 0.697 | 1.075 | 0.191 |
| Job social support | 0.021 | -6.000 | 0.863 | 0.822 | 0.905 | 0.000 |
| Degree of job stress | 31.362 | 0.810 | 10.675 | 0.034 | 3382.175 | 0.420 |
| ***Lifestyle*** |  |  |  |  |  |  |
| Nap at lunchtime |  |  |  |  |  |  |
| yes | 0.157 | -0.210 | 0.966 | 0.702 | 1.330 | 0.834 |
| Alcohol consumption |  |  |  |  |  |  |
| yes | 0.596 | 1.670 | 1.761 | 0.907 | 3.420 | 0.094 |
| Smoking |  |  |  |  |  |  |
| yes | 0.396 | 0.240 | 1.092 | 0.536 | 2.224 | 0.808 |
| Exercise habits |  |  |  |  |  |  |
| yes | 0.295 | -0.380 | 0.881 | 0.457 | 1.697 | 0.704 |
| Exercise frequency (per week) |  |  |  |  |  |  |
| 3-4 times | 0.316 | 0.340 | 1.103 | 0.629 | 1.934 | 0.733 |
| Exercise length (min/time) | 0.004 | 0.290 | 1.001 | 0.994 | 1.009 | 0.775 |
| ***Mental and physical health*** |  |  |  |  |  |  |
| **Resilience total score** | 0.019 | -7.890 | **0.837**** | 0.801 | 0.875 | 0.000 |
| **Adaptive performance** | 0.017 | 9.480 | **1.148**** | 1.116 | 1.181 | 0.000 |
| Social support | 0.040 | -0.610 | 0.863 | 0.899 | 1.057 | 0.539 |
| **Professional identity** | 0.021 | -6.440 | **0.852**** | 0.811 | 0.894 | 0.000 |
| **Bullying** | 0.019 | -5.770 | **0.886**** | 0.850 | 0.923 | 0.000 |
| ***Workplace Violence Exposure*** |  |  |  |  |  |  |
| **Witness of colleagues’ WPV** |  |  |  |  |  |  |
| **yes** | 3.567 | 8.730 | **12.378**** | 7.036 | 21.775 | 0.000 |
| **Witness of colleagues’ physical assault** | 0.267 | 9.780 | **2.666**** | 2.190 | 3.244 | 0.000 |
| **Witness of colleagues’ emotional abuse** | 0.055 | -5.770 | **0.577**** | 0.478 | 0.695 | 0.000 |
| Witness of colleagues’ threatening | 0.090 | 0.000 | 1.000 | 0.839 | 1.191 | 0.996 |
| **Witness of colleagues’ verbal assault** | 0.097 | -2.060 | **0.771*** | 0.602 | 0.987 | 0.039 |
| Witness of colleagues’ sexual assault | 0.142 | -0.180 | 0.974 | 0.732 | 1.296 | 0.856 |

Note:**P＜0.01；*P＜0.05.

**Supplementary Table 4. The protective and risk factors of self-involving emotional abuse by ordered logit model.**

| **Variables** | β | SE | OR | 95% CI of OR | | *p*  value |
| --- | --- | --- | --- | --- | --- | --- |
|  |  |  |  | lower | upper |  |
| ***Demographic characteristics*** |  |  |  |  |  |  |
| **Gender** |  |  |  |  |  |  |
| **female** | 0.107 | -2.27 | **0.712^*^** | 0.531 | 0.955 | 0.023 |
| Age | 0.028 | -0.68 | 0.980 | 0.926 | 1.038 | 0.494 |
| Region |  |  |  |  |  |  |
| urban | 0.112 | 1.64 | 1.170 | 0.969 | 1.413 | 0.102 |
| Education |  |  |  |  |  |  |
| two-year college | 0.172 | -1.21 | 0.761 | 0.488 | 1.186 | 0.227 |
| postgraduate | 0.172 | -1.42 | 0.708 | 0.440 | 1.139 | 0.154 |
| Marital status |  |  |  |  |  |  |
| married | 0.241 | -1.48 | 0.456 | 0.162 | 1.286 | 0.138 |
| others | 0.335 | -0.83 | 0.653 | 0.239 | 1.783 | 0.406 |
| **Religious belief** |  |  |  |  |  |  |
| **yes** | 0.219 | 2.86 | **1.513^**^** | 1.139 | 2.009 | 0.004 |
| Number of household members |  |  |  |  |  |  |
| one person | 0.306 | 0.59 | 1.166 | 0.698 | 1.950 | 0.557 |
| two persons | 0.202 | -0.75 | 0.835 | 0.519 | 1.342 | 0.456 |
| > two persons | 0.208 | -0.53 | 0.883 | 0.557 | 1.400 | 0.597 |
| **Living with parents** |  |  |  |  |  |  |
| **yes** | 0.069 | -2.36 | **0.819^*^** | 0.693 | 0.966 | 0.018 |
| Experience of being left behind |  |  |  |  |  |  |
| yes | 0.096 | 1.21 | 1.111 | 0.937 | 1.316 | 0.226 |
| ***Working Conditions*** |  |  |  |  |  |  |
| Hospital level |  |  |  |  |  |  |
| tertiary III | 0.129 | 0.32 | 1.041 | 0.816 | 1.327 | 0.747 |
| **Occupational exposure** |  |  |  |  |  |  |
| **yes** | 0.127 | 4.08 | **1.434^**^** | 1.206 | 1.705 | 0.000 |
| Working length (hours/week) |  |  |  |  |  |  |
| 41 to 55 hours | 0.115 | 1.15 | 1.125 | 0.920 | 1.374 | 0.251 |
| 56 to 70 hours | 0.172 | 0.35 | 1.058 | 0.769 | 1.454 | 0.730 |
| more than 70 hours | 0.280 | 0.64 | 1.165 | 0.727 | 1.867 | 0.525 |
| Own income per month |  |  |  |  |  |  |
| below China’s national average disposable income | 0.316 | -0.15 | 0.950 | 0.495 | 1.825 | 0.878 |
| above China’s national average disposable income | 0.401 | 0.59 | 1.214 | 0.636 | 2.318 | 0.557 |
| Day shift frequency per week |  |  |  |  |  |  |
| 15 to 22 days | 0.121 | 0.15 | 1.018 | 0.807 | 1.284 | 0.881 |
| > 22 days | 0.126 | -0.76 | 0.899 | 0.683 | 1.184 | 0.449 |
| Day shift length (hours) | 0.055 | -1.18 | 0.933 | 0.832 | 1.047 | 0.238 |
| Night shift frequency per month |  |  |  |  |  |  |
| 2 to 6 times | 0.109 | 0.99 | 1.102 | 0.908 | 1.338 | 0.324 |
| > 6 times | 0.168 | 1.35 | 1.207 | 0.919 | 1.585 | 0.176 |
| Night shift length | 0.036 | 1.27 | 1.045 | 0.977 | 1.117 | 0.203 |
| **Caring patients (no.)** |  |  |  |  |  |  |
| 5-8 | 0.164 | 1.16 | 1.175 | 0.894 | 1.543 | 0.248 |
| 9-12 | 0.158 | 1.46 | 1.209 | 0.937 | 1.561 | 0.145 |
| **>12** | 0.182 | 2.83 | **1.433^**^** | 1.117 | 1.838 | 0.005 |
| Patients’ death (no.) |  |  |  |  |  |  |
| one | 0.136 | 0.33 | 1.044 | 0.808 | 1.348 | 0.742 |
| > one | 0.100 | 0.48 | 1.047 | 0.869 | 1.262 | 0.628 |
| Daily commuting time to work (hours) | 0.072 | 0.39 | 1.027 | 0.896 | 1.178 | 0.700 |
| **Own medical complaints** |  |  |  |  |  |  |
| **yes** | 0.279 | 3.59 | **1.763^**^** | 1.294 | 2.404 | 0.000 |
| **Medical complaints of colleagues** |  |  |  |  |  |  |
| **yes** | 0.075 | -2.1 | **0.827^*^** | 0.692 | 0.987 | 0.036 |
| **Feel a balance between effort and return** |  |  |  |  |  |  |
| unsatisfied | 0.500 | -0.19 | 0.898 | 0.302 | 2.675 | 0.847 |
| normal | 0.315 | -0.86 | 0.666 | 0.264 | 1.681 | 0.390 |
| satisfied | 0.247 | -1.38 | 0.520 | 0.205 | 1.319 | 0.169 |
| **very satisfied** | 0.165 | -2.21 | **0.323^*^** | 0.118 | 0.881 | 0.027 |
| Career prospects |  |  |  |  |  |  |
| unchanged | 0.138 | -0.6 | 0.913 | 0.679 | 1.228 | 0.548 |
| higher than current level | 0.111 | -1.86 | 0.764 | 0.575 | 1.014 | 0.063 |
| **Job demands** | 0.070 | -2.76 | **0.780^**^** | 0.653 | 0.930 | 0.006 |
| **Job control** | 0.049 | -2.08 | **0.891^*^** | 0.800 | 0.993 | 0.038 |
| **Job social support** | 0.014 | -10.35 | **0.844^**^** | 0.817 | 0.871 | 0.000 |
| Degree of job stress | 6.192 | 0.95 | 4.151 | 0.223 | 77.244 | 0.340 |
| ***Lifestyle*** |  |  |  |  |  |  |
| Nap at lunchtime |  |  |  |  |  |  |
| yes | 0.103 | 1.3 | 1.126 | 0.941 | 1.347 | 0.195 |
| Alcohol consumption |  |  |  |  |  |  |
| yes | 0.214 | -0.58 | 0.867 | 0.535 | 1.406 | 0.563 |
| Smoking |  |  |  |  |  |  |
| yes | 0.262 | 0.16 | 1.042 | 0.636 | 1.707 | 0.870 |
| Exercise habits |  |  |  |  |  |  |
| yes | 0.194 | -0.38 | 0.924 | 0.612 | 1.395 | 0.708 |
| Exercise frequency (per week) |  |  |  |  |  |  |
| 3-4 times | 0.164 | -0.46 | 0.921 | 0.651 | 1.305 | 0.645 |
| Exercise length (min/time) | 0.002 | -0.06 | 1.000 | 0.995 | 1.005 | 0.954 |
| ***Mental and physical health*** |  |  |  |  |  |  |
| **Resilience total score** | 0.012 | -12.31 | **0.834^**^** | 0.810 | 0.859 | 0.000 |
| **Adaptive performance** | 0.011 | 14.09 | **1.147^**^** | 1.125 | 1.169 | 0.000 |
| Social support | 0.025 | 0.77 | 1.019 | 0.971 | 1.070 | 0.441 |
| **Professional identity** | 0.013 | -11.87 | **0.834^**^** | 0.809 | 0.859 | 0.000 |
| **Bullying** | 0.012 | -10.64 | **0.861^**^** | 0.838 | 0.885 | 0.000 |
| ***Workplace Violence Exposure*** |  |  |  |  |  |  |
| **Witness of colleagues’ WPV** |  |  |  |  |  |  |
| **yes** | 3.270 | 16.52 | **18.514^**^** | 13.096 | 26.173 | 0.000 |
| Witness of colleagues’ physical assault | 0.060 | 0.34 | 1.020 | 0.910 | 1.144 | 0.730 |
| **Witness of colleagues’ emotional abuse** | 0.090 | 7.93 | **1.571^**^** | 1.405 | 1.757 | 0.000 |
| Witness of colleagues’ threatening | 0.055 | -0.1 | 0.994 | 0.892 | 1.108 | 0.917 |
| **Witness of colleagues’ verbal assault** | 0.048 | -6.18 | **0.617^**^** | 0.529 | 0.719 | 0.000 |
| Witness of colleagues’ sexual assault | 0.094 | 0 | 1.000 | 0.831 | 1.202 | 0.996 |

**Note:** ** p<0.01, * p<0.05

**Supplementary Table 5. The protective and risk factors of self-involving threatening by ordered logit model.**

| **Variables** | β | SE | OR | 95% CI of OR | | *p*  value |
| --- | --- | --- | --- | --- | --- | --- |
|  |  |  |  | lower | upper |  |
| ***Demographic characteristics*** |  |  |  |  |  |  |
| **Gender** |  |  |  |  |  |  |
| **female** | 0.134 | -1.990 | **0.671^*^** | 0.453 | 0.994 | 0.047 |
| Age | 0.041 | -0.170 | 0.993 | 0.915 | 1.078 | 0.868 |
| Region |  |  |  |  |  |  |
| urban | 0.121 | -0.890 | 0.886 | 0.678 | 1.158 | 0.375 |
| Education |  |  |  |  |  |  |
| two-year college | 0.353 | 0.380 | 1.127 | 0.610 | 2.081 | 0.702 |
| postgraduate | 0.370 | 0.270 | 1.095 | 0.564 | 2.125 | 0.790 |
| Marital status |  |  |  |  |  |  |
| married | 0.651 | -0.440 | 0.633 | 0.084 | 4.753 | 0.656 |
| others | 0.574 | -0.560 | 0.567 | 0.078 | 4.122 | 0.575 |
| Religious belief |  |  |  |  |  |  |
| yes | 0.265 | 0.780 | 1.190 | 0.770 | 1.840 | 0.434 |
| Number of household members |  |  |  |  |  |  |
| one person | 0.451 | 0.670 | 1.268 | 0.631 | 2.546 | 0.505 |
| two persons | 0.328 | 0.160 | 1.052 | 0.571 | 1.940 | 0.870 |
| > two persons | 0.250 | -0.680 | 0.812 | 0.443 | 1.486 | 0.499 |
| Living with parents |  |  |  |  |  |  |
| yes | 0.105 | -1.260 | 0.856 | 0.673 | 1.090 | 0.207 |
| Experience of being left behind |  |  |  |  |  |  |
| yes | 0.123 | -0.190 | 0.976 | 0.763 | 1.248 | 0.846 |
| ***Working Conditions*** |  |  |  |  |  |  |
| Hospital level |  |  |  |  |  |  |
| tertiary III | 0.243 | 1.290 | 1.277 | 0.880 | 1.855 | 0.198 |
| Occupational exposure |  |  |  |  |  |  |
| yes | 0.151 | 1.530 | 1.209 | 0.947 | 1.544 | 0.127 |
| **Working length (hours/week)** |  |  |  |  |  |  |
| **41 to 55 hours** | 0.107 | -2.010 | **0.752^*^** | 0.569 | 0.993 | 0.045 |
| 56 to 70 hours | 0.167 | -1.410 | 0.722 | 0.459 | 1.137 | 0.160 |
| more than 70 hours | 0.306 | 0.160 | 1.048 | 0.591 | 1.857 | 0.873 |
| Own income per month |  |  |  |  |  |  |
| below China’s national average disposable income | 0.441 | 0.290 | 1.120 | 0.518 | 2.423 | 0.774 |
| above China’s national average disposable income | 0.427 | 0.240 | 1.098 | 0.513 | 2.351 | 0.809 |
| Day shift frequency per week |  |  |  |  |  |  |
| 15 to 22 days | 0.170 | 0.250 | 1.042 | 0.757 | 1.435 | 0.801 |
| > 22 days | 0.177 | -0.590 | 0.890 | 0.602 | 1.314 | 0.556 |
| Day shift length (hours) | 0.084 | -0.300 | 0.974 | 0.823 | 1.153 | 0.760 |
| Night shift frequency per month |  |  |  |  |  |  |
| 2 to 6 times | 0.165 | 0.960 | 1.147 | 0.866 | 1.519 | 0.339 |
| > 6 times | 0.275 | 1.700 | 1.398 | 0.950 | 2.056 | 0.089 |
| Night shift length | 0.051 | 1.590 | 1.079 | 0.983 | 1.184 | 0.111 |
| **Caring patients (no.)** |  |  |  |  |  |  |
| 5-8 | 0.270 | 1.310 | 1.311 | 0.876 | 1.962 | 0.189 |
| **9-12** | 0.298 | 2.210 | **1.536^*^** | 1.050 | 2.247 | 0.027 |
| **>12** | 0.306 | 2.450 | **1.599^*^** | 1.099 | 2.326 | 0.014 |
| Patients’ death (no.) |  |  |  |  |  |  |
| one | 0.194 | 0.650 | 1.120 | 0.797 | 1.573 | 0.514 |
| > one | 0.122 | -0.740 | 0.904 | 0.694 | 1.178 | 0.456 |
| Daily commuting time to work (hours) | 0.113 | 1.510 | 1.158 | 0.957 | 1.403 | 0.132 |
| **Own medical complaints** |  |  |  |  |  |  |
| **yes** | 0.443 | 3.500 | **2.096^**^** | 1.385 | 3.172 | 0.000 |
| Medical complaints of colleagues |  |  |  |  |  |  |
| yes | 0.153 | 0.950 | 1.136 | 0.873 | 1.479 | 0.343 |
| Feel a balance between effort and return |  |  |  |  |  |  |
| unsatisfied | 1.288 | 0.980 | 1.923 | 0.517 | 7.144 | 0.329 |
| normal | 0.942 | 1.090 | 1.781 | 0.632 | 5.022 | 0.275 |
| satisfied | 0.783 | 0.740 | 1.480 | 0.525 | 4.176 | 0.458 |
| very satisfied | 0.621 | 0.090 | 1.052 | 0.331 | 3.345 | 0.931 |
| Career prospects |  |  |  |  |  |  |
| unchanged | 0.174 | -0.710 | 0.867 | 0.586 | 1.283 | 0.476 |
| higher than current level | 0.141 | -1.580 | 0.739 | 0.508 | 1.075 | 0.114 |
| **Job demands** | 0.104 | -2.340 | **0.708^*^** | 0.531 | 0.945 | 0.019 |
| Job control | 0.075 | -1.470 | 0.882 | 0.746 | 1.042 | 0.141 |
| **Job social support** | 0.019 | -6.940 | **0.861^**^** | 0.825 | 0.898 | 0.000 |
| Degree of job stress | 19.768 | 0.850 | 8.041 | 0.065 | 994.917 | 0.396 |
| ***Lifestyle*** |  |  |  |  |  |  |
| Nap at lunchtime |  |  |  |  |  |  |
| yes | 0.156 | 1.230 | 1.177 | 0.908 | 1.525 | 0.218 |
| Alcohol consumption |  |  |  |  |  |  |
| yes | 0.328 | 0.160 | 1.050 | 0.570 | 1.936 | 0.875 |
| Smoking |  |  |  |  |  |  |
| yes | 0.310 | -0.630 | 0.777 | 0.355 | 1.698 | 0.527 |
| Exercise habits |  |  |  |  |  |  |
| yes | 0.404 | 1.350 | 1.453 | 0.843 | 2.505 | 0.178 |
| Exercise frequency (per week) |  |  |  |  |  |  |
| 3-4 times | 0.165 | -1.470 | 0.709 | 0.449 | 1.120 | 0.141 |
| Exercise length (min/time) | 0.004 | -0.950 | 0.997 | 0.990 | 1.004 | 0.341 |
| ***Mental and physical health*** |  |  |  |  |  |  |
| **Resilience total score** | 0.016 | -9.980 | **0.824^**^** | 0.793 | 0.856 | 0.000 |
| **Adaptive performance** | 0.015 | 11.500 | **1.157^**^** | 1.128 | 1.186 | 0.000 |
| Social support | 0.036 | -0.120 | 0.996 | 0.928 | 1.068 | 0.906 |
| **Professional identity** | 0.018 | -7.430 | **0.853^**^** | 0.818 | 0.889 | 0.000 |
| **Bullying** | 0.017 | -7.490 | **0.866^**^** | 0.834 | 0.900 | 0.000 |
| ***Workplace Violence Exposure*** |  |  |  |  |  |  |
| **Witness of colleagues’ WPV** |  |  |  |  |  |  |
| **yes** | **3.629** | **10.300** | **14.120^**^** | 8.532 | 23.368 | 0.000 |
| Witness of colleagues’ physical assault | 0.078 | 0.390 | 1.030 | 0.888 | 1.195 | 0.696 |
| **Witness of colleagues’ emotional abuse** | **0.049** | **-5.350** | **0.681^**^** | 0.592 | 0.784 | 0.000 |
| **Witness of colleagues’ threatening** | **0.192** | **12.930** | **2.602^**^** | 2.251 | 3.007 | 0.000 |
| **Witness of colleagues’ verbal assault** | **0.068** | **-3.600** | **0.705^**^** | 0.583 | 0.853 | 0.000 |
| Witness of colleagues’ sexual assault | 0.114 | -0.36 | 0.958 | 0.759 | 1.210 | 0.721 |

Note:**P＜0.01；*P＜0.05.

**Supplementary Table 6. The protective and risk factors of self-involving verbal assault by ordered logit model.**

| **Variables** | β | SE | OR | 95% CI of OR | | *p*  value |
| --- | --- | --- | --- | --- | --- | --- |
|  |  |  |  | lower | upper |  |
| ***Demographic characteristics*** |  |  |  |  |  |  |
| **Gender** |  |  |  |  |  |  |
| **female** | 0.493 | 2.100 | **1.786^*^** | 1.040 | 3.069 | 0.036 |
| Age | 0.042 | -1.130 | 0.951 | 0.872 | 1.038 | 0.260 |
| Region |  |  |  |  |  |  |
| urban | 0.151 | 0.370 | 1.054 | 0.797 | 1.395 | 0.712 |
| Education |  |  |  |  |  |  |
| two-year college | 0.550 | 1.050 | 1.479 | 0.714 | 3.065 | 0.292 |
| postgraduate | 0.664 | 1.320 | 1.683 | 0.777 | 3.648 | 0.187 |
| Marital status |  |  |  |  |  |  |
| married | 0.470 | -0.760 | 0.466 | 0.064 | 3.367 | 0.449 |
| others | 0.750 | -0.270 | 0.769 | 0.114 | 5.201 | 0.788 |
| **Religious belief** |  |  |  |  |  |  |
| **yes** | 0.369 | 2.450 | **1.700^*^** | 1.111 | 2.600 | 0.014 |
| **Number of household members** |  |  |  |  |  |  |
| one person | 0.233 | -1.100 | 0.690 | 0.355 | 1.338 | 0.272 |
| two persons | 0.221 | -0.890 | 0.776 | 0.445 | 1.355 | 0.373 |
| **> two persons** | 0.142 | -2.380 | **0.523^*^** | 0.307 | 0.892 | 0.017 |
| Living with parents |  |  |  |  |  |  |
| yes | 0.133 | 0.140 | 1.018 | 0.788 | 1.316 | 0.889 |
| Experience of being left behind |  |  |  |  |  |  |
| yes | 0.149 | 0.610 | 1.087 | 0.831 | 1.421 | 0.545 |
| ***Working Conditions*** |  |  |  |  |  |  |
| Hospital level |  |  |  |  |  |  |
| tetirary III | 0.145 | -1.470 | 0.754 | 0.518 | 1.098 | 0.141 |
| **Occupational exposure** |  |  |  |  |  |  |
| **yes** | 0.219 | 3.250 | **1.572^**^** | 1.196 | 2.067 | 0.001 |
| Working length (hours/week) |  |  |  |  |  |  |
| 41 to 55 hours | 0.214 | 1.330 | 1.254 | 0.897 | 1.753 | 0.185 |
| 56 to 70 hours | 0.300 | 0.690 | 1.189 | 0.725 | 1.951 | 0.492 |
| more than 70 hours | 0.447 | 0.870 | 1.336 | 0.693 | 2.573 | 0.387 |
| Own income per month |  |  |  |  |  |  |
| below China’s national average disposable income | 0.290 | -0.850 | 0.704 | 0.314 | 1.577 | 0.393 |
| above China’s national average disposable income | 0.352 | -0.340 | 0.874 | 0.397 | 1.923 | 0.737 |
| Day shift frequency per week |  |  |  |  |  |  |
| 15 to 22 days | 0.218 | 1.010 | 1.201 | 0.842 | 1.715 | 0.312 |
| > 22 days | 0.233 | 0.360 | 1.081 | 0.709 | 1.649 | 0.718 |
| Day shift length (hours) | 0.081 | -1.390 | 0.880 | 0.735 | 1.053 | 0.163 |
| Night shift frequency per month |  |  |  |  |  |  |
| 2 to 6 times | 0.180 | 0.760 | 1.130 | 0.826 | 1.544 | 0.445 |
| > 6 times | 0.208 | -0.340 | 0.925 | 0.595 | 1.439 | 0.731 |
| Night shift length | 0.055 | 0.560 | 1.030 | 0.928 | 1.144 | 0.576 |
| Caring patients (no.) |  |  |  |  |  |  |
| 5-8 | 0.328 | 1.910 | 1.512 | 0.988 | 2.314 | 0.057 |
| 9-12 | 0.256 | 1.010 | 1.233 | 0.821 | 1.853 | 0.313 |
| >12 | 0.245 | 1.000 | 1.223 | 0.825 | 1.812 | 0.316 |
| Patients’ death (no.) |  |  |  |  |  |  |
| one | 0.263 | 1.650 | 1.373 | 0.943 | 1.999 | 0.099 |
| > one | 0.139 | -0.420 | 0.940 | 0.704 | 1.257 | 0.678 |
| Daily commuting time to work (hours) | 0.120 | 0.960 | 1.109 | 0.898 | 1.370 | 0.336 |
| Own medical complaints |  |  |  |  |  |  |
| yes | 0.330 | 0.950 | 1.278 | 0.770 | 2.120 | 0.343 |
| Medical complaints of colleagues |  |  |  |  |  |  |
| yes | 0.134 | -0.420 | 0.942 | 0.713 | 1.246 | 0.676 |
| Feel a balance between effort and return |  |  |  |  |  |  |
| unsatisified | 0.431 | -0.830 | 0.463 | 0.075 | 2.871 | 0.408 |
| normal | 0.694 | 0.180 | 1.117 | 0.330 | 3.774 | 0.859 |
| satisified | 0.813 | 0.430 | 1.307 | 0.386 | 4.425 | 0.667 |
| very satisfied | 0.683 | -0.020 | 0.986 | 0.254 | 3.830 | 0.984 |
| **Career prospects** |  |  |  |  |  |  |
| **unchanged** | 0.140 | -2.040 | **0.640^*^** | 0.416 | 0.983 | 0.042 |
| higher than current level | 0.134 | -2.100 | 0.648 | 0.432 | 0.972 | 0.036 |
| Job demands | 0.136 | -1.150 | 0.827 | 0.598 | 1.142 | 0.249 |
| Job control | 0.085 | -1.180 | 0.893 | 0.741 | 1.078 | 0.239 |
| **Job social support** | 0.021 | -6.590 | **0.852^**^** | 0.812 | 0.893 | 0.000 |
| Degree of job stress | 2.949 | 0.020 | 1.067 | 0.005 | 240.617 | 0.981 |
| ***Lifestyle*** |  |  |  |  |  |  |
| Nap at lunchtime |  |  |  |  |  |  |
| yes | 0.139 | -0.270 | 0.962 | 0.724 | 1.277 | 0.787 |
| Alcohol consumption |  |  |  |  |  |  |
| yes | 0.301 | -0.480 | 0.843 | 0.418 | 1.699 | 0.632 |
| Smoking |  |  |  |  |  |  |
| yes | 0.505 | 0.680 | 1.301 | 0.608 | 2.784 | 0.498 |
| Exercise habits |  |  |  |  |  |  |
| yes | 0.326 | -0.150 | 0.950 | 0.484 | 1.862 | 0.880 |
| Exercise frequency (per week) |  |  |  |  |  |  |
| 3-4 times | 0.268 | -0.210 | 0.942 | 0.539 | 1.647 | 0.834 |
| Exercise length (min/time) | 0.004 | 0.390 | 1.001 | 0.994 | 1.009 | 0.694 |
| ***Mental and physical health*** |  |  |  |  |  |  |
| **Resilience total score** | 0.018 | -7.390 | **0.853^**^** | 0.818 | 0.890 | 0.000 |
| **Adaptive performance** | 0.016 | 8.390 | **1.130^**^** | 1.098 | 1.163 | 0.000 |
| Social support | 0.037 | -1.310 | 0.950 | 0.881 | 1.026 | 0.191 |
| **Professional identity** | 0.020 | -6.160 | **0.865^**^** | 0.826 | 0.906 | 0.000 |
| **Bullying** | 0.020 | -4.170 | **0.912^**^** | 0.873 | 0.952 | 0.000 |
| **Workplace Violence Exposure** |  |  |  |  |  |  |
| **Witness of colleagues’ WPV** |  |  |  |  |  |  |
| **yes** | 6.791 | 9.140 | **20.540^**^** | 10.744 | 39.268 | 0.000 |
| Witness of colleagues’ physical assault | 0.087 | -0.090 | 0.992 | 0.835 | 1.178 | 0.927 |
| Witness of colleagues’ emotional abuse | 0.078 | -0.830 | 0.933 | 0.792 | 1.099 | 0.408 |
| Witness of colleagues’ threatening | 0.068 | -1.880 | 0.863 | 0.740 | 1.006 | 0.059 |
| **Witness of colleagues’ verbal assault** | 0.166 | 6.660 | **1.828^**^** | 1.531 | 2.184 | 0.000 |
| Witness of colleagues’ sexual assault | 0.125 | 0.540 | 1.066 | 0.846 | 1.342 | 0.589 |

**Note:** ** p<0.01, * p<0.05

**Supplementary Table 7. The protective and risk factors of self-involving sexual assault by ordered logit model.**

| **Variables** | β | SE | OR | 95% CI of OR | | *p*  value |
| --- | --- | --- | --- | --- | --- | --- |
|  |  |  |  | lower | upper |  |
| ***Demographic characteristics*** |  |  |  |  |  |  |
| Gender |  |  |  |  |  |  |
| female | 0.713 | 1.340 | 1.736 | 0.776 | 3.884 | 0.179 |
| Age | 0.064 | 0.590 | 1.037 | 0.919 | 1.170 | 0.557 |
| Region |  |  |  |  |  |  |
| urban | 0.233 | 0.920 | 1.196 | 0.816 | 1.753 | 0.358 |
| Education |  |  |  |  |  |  |
| two-year college | 4.976 | 1.710 | 5.184 | 0.790 | 34.022 | 0.086 |
| postgraduate | 4.618 | 1.610 | 4.766 | 0.714 | 31.834 | 0.107 |
| **Marital status** |  |  |  |  |  |  |
| **married** | 0.094 | -2.270 | **0.088^*^** | 0.011 | 0.717 | 0.023 |
| others | 0.255 | -1.360 | 0.253 | 0.035 | 1.822 | 0.173 |
| Religious belief |  |  |  |  |  |  |
| yes | 0.376 | 0.190 | 1.069 | 0.537 | 2.129 | 0.849 |
| Number of household members |  |  |  |  |  |  |
| one person | 0.612 | 0.230 | 1.131 | 0.392 | 3.264 | 0.820 |
| two persons | 0.567 | 0.350 | 1.184 | 0.463 | 3.027 | 0.725 |
| > two persons | 0.368 | -0.530 | 0.777 | 0.307 | 1.966 | 0.594 |
| Living with parents |  |  |  |  |  |  |
| yes | 0.163 | -0.840 | 0.851 | 0.585 | 1.238 | 0.399 |
| Experience of being left behind |  |  |  |  |  |  |
| yes | 0.190 | 0.210 | 1.039 | 0.726 | 1.487 | 0.835 |
| **Working Conditions** |  |  |  |  |  |  |
| Hospital level |  |  |  |  |  |  |
| tetirary III | 0.243 | -0.630 | 0.831 | 0.469 | 1.473 | 0.526 |
| Occupational exposure |  |  |  |  |  |  |
| yes | 0.271 | 1.770 | 1.407 | 0.964 | 2.053 | 0.076 |
| Working length (hours/week) |  |  |  |  |  |  |
| 41 to 55 hours | 0.222 | -0.280 | 0.936 | 0.588 | 1.491 | 0.781 |
| 56 to 70 hours | 0.386 | 0.260 | 1.094 | 0.548 | 2.185 | 0.798 |
| more than 70 hours | 0.652 | 1.120 | 1.583 | 0.706 | 3.550 | 0.265 |
| Own income per month |  |  |  |  |  |  |
| below China’s national average disposable income | 0.299 | -1.000 | 0.616 | 0.237 | 1.597 | 0.319 |
| above China’s national average disposable income | 0.312 | -0.860 | 0.671 | 0.270 | 1.669 | 0.391 |
| Day shift frequency per week |  |  |  |  |  |  |
| 15 to 22 days | 0.257 | 0.050 | 1.012 | 0.616 | 1.664 | 0.962 |
| > 22 days | 0.376 | 1.010 | 1.329 | 0.764 | 2.312 | 0.314 |
| Day shift length (hours) | 0.118 | -0.540 | 0.934 | 0.728 | 1.197 | 0.587 |
| Night shift frequency per month |  |  |  |  |  |  |
| 2 to 6 times | 0.248 | 0.310 | 1.073 | 0.683 | 1.687 | 0.759 |
| > 6 times | 0.398 | 0.930 | 1.322 | 0.733 | 2.385 | 0.353 |
| Night shift length | 0.084 | -0.490 | 0.958 | 0.806 | 1.139 | 0.626 |
| Caring patients (no.) |  |  |  |  |  |  |
| 5-8 | 0.510 | 1.580 | 1.635 | 0.887 | 3.013 | 0.115 |
| 9-12 | 0.354 | 0.470 | 1.156 | 0.634 | 2.108 | 0.637 |
| >12 | 0.301 | 0.130 | 1.037 | 0.587 | 1.832 | 0.900 |
| Patients’ death (no.) |  |  |  |  |  |  |
| one | 0.401 | 1.240 | 1.421 | 0.817 | 2.472 | 0.214 |
| > one | 0.237 | 0.460 | 1.104 | 0.725 | 1.681 | 0.644 |
| Commuting (hours/day) | 0.154 | 0.210 | 1.033 | 0.770 | 1.384 | 0.830 |
| **Own medical complaints** |  |  |  |  |  |  |
| **yes** | 0.759 | 2.250 | **2.186^*^** | 1.108 | 4.316 | 0.024 |
| Medical complaints of colleagues |  |  |  |  |  |  |
| yes | 0.168 | -1.020 | 0.810 | 0.540 | 1.216 | 0.310 |
| Feel a balance between effort and return |  |  |  |  |  |  |
| unsatisified | 0.362 | -1.000 | 0.300 | 0.028 | 3.199 | 0.319 |
| normal | 0.613 | 0.740 | 1.387 | 0.583 | 3.297 | 0.459 |
| satisified | 0.506 | 0.340 | 1.160 | 0.493 | 2.727 | 0.734 |
| very satisified | 0.620 | 0.000 | 1.003 | 0.298 | 3.370 | 0.996 |
| **Career prospects** |  |  |  |  |  |  |
| **unchanged** | 0.154 | -2.210 | **0.522^*^** | 0.294 | 0.930 | 0.027 |
| **higher than current level** | 0.138 | -2.500 | **0.503^*^** | 0.294 | 0.863 | 0.013 |
| Job demands | 0.400 | 0.150 | 1.060 | 0.505 | 2.222 | 0.878 |
| Job control | 0.146 | -1.740 | 0.693 | 0.459 | 1.047 | 0.082 |
| **Job social support** | 0.029 | -4.410 | **0.864^**^** | 0.809 | 0.922 | 0.000 |
| Degree of job stress | 0.021 | -0.880 | 0.003 | **<0.0001** | 1049.371 | 0.377 |
| ***Lifestyle*** |  |  |  |  |  |  |
| Nap at lunchtime |  |  |  |  |  |  |
| yes | 0.195 | -0.220 | 0.957 | 0.642 | 1.426 | 0.827 |
| Alcohol consumption |  |  |  |  |  |  |
| yes | 0.604 | 0.270 | 1.151 | 0.411 | 3.221 | 0.789 |
| Smoking |  |  |  |  |  |  |
| yes | 0.337 | -1.020 | 0.505 | 0.136 | 1.866 | 0.305 |
| Exercise habits |  |  |  |  |  |  |
| yes | 0.391 | -0.170 | 0.930 | 0.408 | 2.122 | 0.863 |
| Exercise frequency (per week) |  |  |  |  |  |  |
| 3-4 times | 0.345 | -0.280 | 0.896 | 0.422 | 1.904 | 0.776 |
| Exercise length (min/time) | 0.005 | -0.310 | 0.998 | 0.989 | 1.008 | 0.754 |
| ***Mental and physical health*** |  |  |  |  |  |  |
| **Resilience total score** | 0.025 | -6.370 | **0.826^**^** | 0.778 | 0.876 | 0.000 |
| **Adaptive performance** | 0.022 | 8.550 | **1.172^**^** | 1.130 | 1.215 | 0.000 |
| Social support | 0.048 | -1.560 | 0.922 | 0.832 | 1.021 | 0.120 |
| **Professional identity** | 0.027 | -4.780 | **0.863^**^** | 0.813 | 0.917 | 0.000 |
| **Bullying** | 0.024 | -4.400 | **0.886^**^** | 0.839 | 0.935 | 0.000 |
| ***Workplace Violence Exposure*** |  |  |  |  |  |  |
| **Witness of colleagues’ WPV** |  |  |  |  |  |  |
| **yes** | 16.414 | 6.270 | **30.211^**^** | 10.416 | 87.624 | 0.000 |
| Witness of colleagues’ physical assault | 0.099 | -1.730 | 0.810 | 0.638 | 1.029 | 0.084 |
| Witness of colleagues’ emotional abuse | 0.094 | -1.230 | 0.876 | 0.711 | 1.081 | 0.218 |
| Witness of colleagues’ threatening | 0.099 | -0.580 | 0.941 | 0.766 | 1.155 | 0.562 |
| Witness of colleagues’ verbal assault | 0.108 | -1.260 | 0.852 | 0.664 | 1.093 | 0.207 |
| **Witness of colleagues’ sexual assault** | 0.439 | 6.680 | **2.825^**^** | 2.083 | 3.831 | 0.000 |

**Note:** ** p<0.01, * p<0.05

Regarding the reliability and validity of the scales used in this study, we have summarized the psychometric properties from previous studies as follows: The Chinese version of the Job Content Questionnaire (JCQ) demonstrated acceptable internal consistency, with Cronbach's alpha coefficients ranging from 0.70 to 0.88 across its subscales, and confirmatory factor analysis supported its construct validity (Li et al., 2004). This scale has also been widely used in subsequent studies, such as Lin et al. (2025). For the Adaptive Performance Scale (APS), previous research has established its strong psychometric properties, including good internal reliability (e.g., Cronbach's alpha ranging from 0.86 to 0.91) and construct validity through confirmatory factor analysis (Charbonnier-Voirin & Roussel, 2012; Marques-Quinteiro et al., 2015). The Social Support Rating Scale (SSRS) showed good test-retest reliability (r = 0.78) and internal consistency (Cronbach's alpha = 0.70), with factor analysis confirming its three-factor structure (Reis et al., 2011). Concerning the Professional Identity Scale for Nurses (PISN), a systematic review by Matthews et al. (2019) indicated that while several professional identity measures exist, many require further validation, though the PISN has shown acceptable reliability in preliminary studies. Finally, the Negative Acts Questionnaire (NAQ), particularly the Revised version, has demonstrated high internal consistency (Cronbach's alpha typically between 0.85 and 0.90) and robust construct validity in discriminating between bullied and non-bullied groups, as confirmed in a systematic review and meta-analysis (Serafin et al., 2020).

References

1. Li, J., et al., *Psychometric evaluation of the Chinese (mainland) version of Job Content Questionnaire: a study in university hospitals.* Ind Health, 2004. **42**(2): p. 260-7.

2. Lin, K.H., C.C. Hsu, and K.Y. Lin, *Job stress and burnout among hospital administrative staff: a cross-sectional study.* Sci Rep, 2025. **15**(1): p. 31064.

3. Marques-Quinteiro, P., et al., *Measuring adaptive performance in individuals and teams.* Team Performance Management, 2015. **21**(7/8): p. 339-360.

4. Charbonnier-Voirin, A. and P. Roussel, *Adaptive Performance: A New Scale to Measure Individual Performance in Organizations.* Canadian Journal of Administrative Sciences / Revue Canadienne des Sciences de l'Administration, 2012. **29**(3): p. 280-293.

5. Reis, M.S., R.S. Reis, and P.C. Hallal, *Validity and reliability of a physical activity social support assessment scale.* Rev Saude Publica, 2011. **45**(2): p. 294-301.

6. Matthews, J., A. Bialocerkowski, and M. Molineux, *Professional identity measures for student health professionals – a systematic review of psychometric properties.* BMC Medical Education, 2019. **19**(1): p. 308.

7. Serafin, L., N. Sak-Dankosky, and B. Czarkowska-Pączek, *Bullying in nursing evaluated by the Negative Acts Questionnaire-Revised: A systematic review and meta-analysis.* J Adv Nurs, 2020. **76**(6): p. 1320-1333.

8. Chen, W., et al., *Psychometric properties of the Chinese version of the Resilience Scale (RS-14): Preliminary results.* PLoS One, 2020. **15**(10): p. e0241606.
